# Improved polygenic risk prediction for alzheimer’s disease and related dementias using deep learning: age and *APOE*-stratified analysis

**DOI:** 10.1186/s13195-026-02011-w

**Published:** 2026-03-12

**Authors:** Shayan Mostafaei, Daniel Wikström Shemer, Jonathan K. L. Mak, Ida K. Karlsson, Sara Hägg

**Affiliations:** 1 Department of Medical Epidemiology and Biostatistics, Karolinska Institutet, Stockholm, Sweden; 2 Department of Pharmacology and Pharmacy, Li Ka Shing Faculty of Medicine, The University of Hong Kong, Hong Kong, China

**Keywords:** Dementia, Alzheimer Disease, Polygenic Risk Scores, Apolipoprotein E (*APOE*), Deep learning, UK Biobank

## Abstract

**Background:**

Alzheimer’s disease and related dementias (ADRD) are complex, polygenic conditions with substantial public health impact. Accurate genetic risk prediction may enable earlier identification and stratification of individuals at elevated risk.

**Objective:**

To evaluate the predictive performance of polygenic risk scores (PRS) for ADRD using a Bayesian variational autoencoders approach and to assess the modifying effects of age and *APOE* genotype on model performance.

**Methods:**

We analyzed data from 276,566 unrelated individuals of European ancestry in the UK Biobank, with a median follow-up of 9.19 years. PRS and polygenic hazard scores (PHS) were constructed using genome-wide association study summary statistics, with PHS incorporating age-at-onset information. Three PRS methods were compared: DDML (Bayesian variational autoencoders), SBayesR (Bayesian multiple regression), and clumping and thresholding (C + T). Models were stratified by age and *APOE* genotype. Predictive performance was evaluated using time-dependent AUC, C-index, and hazard ratios (HRs), using a prespecified 2:1 training/testing split with identical ADRD case proportion across splits. All primary results are based on covariate-adjusted models, incorporating PRS together with age, sex, and 10 genetic principal components, and *APOE* genotype where indicated. Classification performance was compared between individuals in the top and bottom PRS quartiles to assess stratified risk.

**Results:**

Among the participants (mean age 56.8 ± 8.0 years; 46.7% male), 1,328 (0.48%) developed ADRD. In covariate-adjusted models, DDML_PRS achieved the highest predictive accuracy (AUC = 0.847) in individuals aged 65–70 years. PHS models showed peak performance at 7 years of follow-up. DDML_PRS significantly improved classification in *APOE-ε4* carriers aged ≥ 65 years and outperformed other models across ADRD subtypes. Significant interactions were observed between PRS performance, age, and *APOE* genotype.

**Conclusion:**

The DDML_PRS framework showed consistently higher discrimination than standard PRS baselines in this UK Biobank setting, particularly in older adults and *APOE-ε4* carriers, supporting its potential for individualized ADRD risk stratification. However, the observed classification accuracy remains modest, limiting immediate clinical utility and underscoring the need for external replication and multi-modal validation (e.g., biomarkers and clinical adjudication) to translate these predictive gains into practical early detection strategies.

**Supplementary Information:**

The online version contains supplementary material available at 10.1186/s13195-026-02011-w.

## Introduction

Alzheimer’s disease and related dementias (ADRD) are progressive neurological disorders characterized by a decline in cognitive functions, including memory, reasoning, and communication skills. Alzheimer’s disease (AD) is the most common type of ADRD, accounting for 60–80% of cases [1]. AD is characterized by the presence of beta-amyloid plaques and tau tangles in the brain, which lead to neuronal injury and the subsequent loss of cognitive abilities [2]. The prevalence of ADRD is significant and continues to rise as the global population ages. According to recent estimates, approximately 50 million people worldwide are living with dementia [3], and this number is projected to triple by 2050, placing an escalating burden on individuals, families, and healthcare systems [4].

Late-onset AD, defined by age at onset of 65 years or older, is a complex genetic disorder with an estimated heritability of 60% to 80% [5]. The most significant genetic risk factor for late-onset AD is the apolipoprotein E (*APOE*) gene. *APOE*, which encodes the brain’s primary cholesterol transporter, has three common alleles: *ε2*, *ε3*, and *ε4*. The *ε4* allele is associated with an increased risk of developing AD, with odds ratios of approximately 3 for heterozygotes (one *ε4* allele) and 8 to 12 for homozygotes (two *ε4* alleles) compared to individuals with the *ε3/ε3* genotype [6, 7]. However, in recent years, polygenic risk scores (PRS) have emerged as a promising tool for quantifying genetic susceptibility to ADRD [8]. Alzheimer’s disease, particularly its late-onset form, is a polygenic disorder, meaning it is influenced by many genetic variants [9, 10]. PRS are calculated by aggregating the effects of these genetic variants, identified through genome-wide association studies (GWAS), and are used to estimate an individual’s genetic predisposition to developing ADRD [8].

Although most GWAS focus on late-onset AD, this study includes both early- and late-onset ADRD cases to enable a broader evaluation of PRS performance across the disease spectrum. Many early-onset cases, particularly those without known monogenic mutations, may still share polygenic risk factors with late-onset AD. In our cohort, a small subset of individuals was classified as early-onset dementia cases, the majority of whom were diagnosed with AD or with mixed, unspecified, or other dementia subtypes. While some early-onset cases may represent distinct conditions such as frontotemporal dementia, their inclusion enhances the generalizability of our findings.

Although PRS can aggregate genetic risk across multiple loci, their clinical utility remains limited due to insufficient predictive power at preclinical stages [8]. Additionally, there is ongoing debate about whether PRS offer superior predictive accuracy compared to the *APOE* genotype alone, especially considering factors such as age and sex, which significantly influence disease risk [8]. Furthermore, many PRS are derived from case-control GWAS, which may not adequately capture age-at-onset information, potentially limiting their utility for early detection [11].

In this study, we leverage the UK Biobank (UKB) to evaluate the performance of a Bayesian variational autoencoders (VAEs)–based polygenic risk scores framework (DDML_PRS) for ADRD risk prediction. This approach combines GWAS-informed Bayesian regularization with latent representation learning to derive a continuous PRS from a predefined set of ADRD-associated variants. Importantly, our primary analyses focus on covariate-adjusted models that integrate PRS with age, sex, and *APOE* genotype, reflecting realistic epidemiological and clinical prediction settings. We compare DDML_PRS with two widely used PRS methods, SBayesR and clumping and thresholding (C + T), and assess performance across age strata and *APOE* genotypes. Although sex was included as a covariate in the models, no significant sex-specific effects were observed, and thus sex-stratified analysis was not the primary focus. This stratified approach aims to improve early detection and individualized risk assessment, supporting the potential clinical translation of PRS-based models for ADRD.

## Methods

### Study design and population

This population-based prospective cohort study includes half a million genotyped individuals aged 37 to 73, with a median follow-up of 9.1 years, recruited across the UK between 2006 and 2010. The UKB contains genetic and health data from participants in the UK [12, 13], enabling in-depth analysis of PRS to predict ADRD outcomes. Diagnostic events of ADRD were ascertained based on the International Classification of Diseases, 10th Revision (ICD-10) codes obtained from hospital inpatient and death registry records (F00, F01, F02, F03, G30, G31.0, G31.1, G31.8). These codes have been previously validated in the UKB, with a positive predictive value of approximately 85% when derived from hospital and mortality data [14]. Participants with a diagnosis or a self-reported history of neurological disorders before baseline were excluded. Exclusions were based on ICD-10 Chapter VI codes (G00–G99), excluding ADRD-related codes, and self-reported conditions from UKB Data-Field 20,002, including Parkinson’s disease, multiple sclerosis, epilepsy, and other neurodegenerative disorders.

The UKB study was approved by the North West Multi-Centre Research Ethics Committee and the specific research aim was further approved by the Swedish Ethical Review Authority. All participants provided written informed consent. We obtained fully de-identified data. Our study adheres to the tenets of the Declaration of Helsinki. This study followed the STROBE reporting guideline for observational studies.

### Genetic and phenotypic data

This study used the extensive genetic resources of the UKB, incorporating imputed dosage data from 488,000 individuals and approximately 96 million genetic variants. *APOE* genotypes and other missing single-nucleotide polymorphisms (SNPs) were imputed using IMPUTE4 software, with reference panels from the Haplotype Reference Consortium (HRC) and the UK10K project [15]. To focus on European ancestry, individuals self-identifying as “White British” were selected and then applied a k-means clustering approach (k = 4) based on the first 10-PCs (principal components) of the genetic data to confirm ancestry [16]. Although UKB provides a PC-filtered ancestry field, we used this two-step approach to ensure stricter control of residual population structure and improve PRS accuracy. To further account for residual population structure within the European ancestry subset, the first 10 genetic PCs were included as covariates in predictive models. This restriction was applied to reduce population stratification and improve the accuracy of PRS evaluation. After further excluding those with any degree of kinship (kinship coefficient > 0.0884) [17], we included 276,566 unrelated individuals of White British/European ancestry in our analyses. Kinship was determined using UK Biobank’s kinship inference data (Data-Field 22021). The flow chart for selecting the UKB participants is shown in Supplementary Fig. 1. For downstream analyses, imputed dosages were converted to hard-call format using a zero threshold, meaning only genotype calls with posterior probability = 1.0 were retained to maximize confidence in variant calling. This conservative approach increases missingness for ambiguous calls, so we applied stringent quality control measures: variants with > 5% missingness after hard-call conversion were excluded, as were individuals with > 5% missing genotype data. Additionally, we removed rare variants with a minor allele frequency below 1%, variants with an imputation info score below 0.8, and those deviating significantly from Hardy-Weinberg equilibrium (*P* < 10⁻¹⁰) in the non-event group [18, 19]. After quality control, a total of 13,367,000 genetic variants were retained for analysis.

To assess the influence of PRSs on the *APOE* locus, we calculated PRSs both with and without including the *APOE* region. We defined the *APOE* region as chr19:44–46 Mb (GRCh37/hg19). For analyses labelled “without *APOE* region”, we excluded all SNPs within this window prior to PRS construction and recomputed the PRS using the remaining variants only. Analyses labelled “with *APOE* region” retained all variants, including those in this window. This comparison allowed us to evaluate the added predictive value of genome-wide polygenic risk beyond the well-established effect of *APOE*, and to determine whether PRS can provide meaningful risk stratification independently of *APOE* genotype. For subgroup and interaction analyses involving *APOE*, participants were classified as *APOE-ε4* carriers (*ε2/ε4*, *ε3/ε4*, *ε4/ε4*) or non-carriers (*ε2/ε2*, *ε2/ε3*, *ε3/ε3*). *APOE ε2/ε3/ε4* genotypes were derived from rs429358 and rs7412.

Seven UKB phenotypes were analyzed. Four of the phenotypes were categorical variables: ADRD status, *APOE-ε4* carrier, sex, and ADRD subtypes. The continuous variables were age at baseline assessment, follow-up time, and the time to ADRD diagnosis. Follow-up time was defined as the duration from baseline assessment to the earliest of ADRD diagnosis, death, or end of follow-up on April 1, 2018. Time to ADRD diagnosis was calculated only for participants who developed ADRD and represents the time from baseline to the first recorded diagnosis [14, 20]. Early-onset ADRD was defined as a first diagnosis before age 65, and late-onset ADRD as a first diagnosis at age 65 or older. ADRD cases were ascertained from linked hospital inpatient and death-registry ICD-10 codes using UKB first-occurrence fields (Data-Fields 42019, 42023, 42025, 130837, 130839, 130841, 130843). In our analysis, we defined subtype groups as: Alzheimer’s disease (G30, F00), vascular dementia (F01), and other/unspecified or mixed dementias (F02, F03, G31.0, G31.1, G31.8). Controls were defined as individuals without any recorded ADRD diagnosis during follow-up.

### GWAS summary statistics and PRS construction

We selected 80 independent SNPs (including the *APOE* locus) that were reported as genome-wide significant in the large-scale GWAS by Bellenguez et al. [9], which identified 83 SNPs across 75 risk loci associated with Alzheimer’s disease and related dementias [9]. Of these, 80 SNPs had available effect size estimates in UK Biobank–excluded GWAS summary statistics from Jansen et al. [21], obtained directly from the authors. Because these SNPs were originally selected based on genome-wide significance in Bellenguez et al. [9], not all selected variants remained genome-wide significant in the UK Biobank–excluded dataset. Within our Bayesian framework, variants with weaker evidence contribute proportionally less through priors with smaller effect sizes and larger uncertainty. This dataset excludes UKB participants and was obtained directly from the authors. It includes clinically diagnosed AD cases and controls from contributing cohorts such as SATSA, IGAP, and ADSP. We used the effect sizes and standard errors from this dataset to weight the selected SNPs in our polygenic risk score construction. These 80 SNPs were used specifically in the DDML model. In contrast, the SBayesR method incorporated approximately one million HapMap3 SNPs using Bayesian multiple regression, and the C + T method included a broader set of variants selected. We leveraged these summary statistics as weights for constructing PRS. To compare the various PRS approaches, we applied three different methods in the UKB participants: (1) Bayesian variational autoencoder (DDML) method; (2) SBayesR, a method that apply Bayesian multiple regression; and (3) the traditional clumping and thresholding (C + T) method. To provide a fair benchmark, we trained a logistic regression model using the same 80 SNPs on the UKB training split. The model was evaluated on the same test set used for DDML_PRS, and no covariates were included.

#### Bayesian variational autoencoders

We employed a DDML approach to construct PRS. The model was developed using individual-level genotype data from 276,566 participants and GWAS summary statistics for 80 selected SNPs. To ensure robust evaluation, the dataset was randomly split into a training set (two-thirds of the sample; *N* = 184,378, including 885 ADRD cases and 183,493 controls) and a testing set (one-third; *N* = 92,188, including 443 ADRD cases and 91,745 controls), preserving the same proportion of ADRD cases across splits. The autoencoder architecture consisted of an encoder with three fully connected layers (512 → 256 → 128 units) using ReLU activation, followed by a 50-dimensional latent space implemented via the reparameterization trick, and a symmetric decoder mirroring the encoder structure. This architecture was pre-specified based on prior applications of variational autoencoders in large-scale genomic modeling [22–24]. In particular, Vivek et al. [24] applied moderately deep VAEs to genome-wide SNP data for dementia prediction, and Li et al. [22, 25] demonstrated that similar architectures effectively capture higher-order and non-linear genetic representations in polygenic trait modeling. These studies informed our choice of network depth and latent dimensionality, balancing representational capacity and interpretability. Although the input dimensionality was modest (80 SNPs), a moderately deep encoder was used to flexibly model correlated and non-linear genetic effects, while effective model complexity was constrained through Bayesian regularization and early stopping. GWAS summary statistics were incorporated as Bayesian priors within the VAEs framework. Effect size estimates from UK Biobank–excluded GWAS summary statistics were used to define prior means, and corresponding standard errors were used to define prior variances. These priors were incorporated through the Kullback–Leibler (KL) divergence term of the loss function, encouraging biologically plausible latent representations while limiting overfitting. KL divergence was linearly annealed from 0 to 1 over the first 20 training epochs to stabilize optimization.

Model architecture and training hyperparameters were fixed a priori based on established VAEs practices in genomics and UKB–scale data [22–24]. Training used the Adam optimizer (learning rate = 0.001, batch size = 256) with a combined reconstruction (mean squared error) and KL divergence loss. Early stopping with a patience of 10 epochs was applied based on validation ELBO, using a 10% internal validation subset drawn exclusively from the training data. No dropout or explicit weight decay was applied, as regularization was provided through Bayesian priors and early stopping. No alternative architectures were evaluated, and no grid or random hyperparameter search was performed. A small number of diagnostic pilot runs (*n* = 2), restricted entirely to the training data, were conducted prior to final model fitting to verify numerical stability and convergence and to assess sensitivity to prior strength (informative GWAS-based priors versus weakly informative priors). These pilot checks did not alter the prespecified architecture or training hyperparameters and were not used for model selection or performance optimization. The independent test set was not used for tuning, early stopping decisions, or model selection and was reserved exclusively for final evaluation. We emphasize that this robustness analysis was intended to assess training stability rather than to optimize performance. The posterior mean of the latent variables was aggregated to produce a single continuous DDML_PRS value for each individual. The DDML model was trained using genotype data only; covariates (age, sex, genetic principal components, and *APOE* genotype where indicated) were incorporated only in downstream regression and survival models. To assess reproducibility, the prespecified final model was retrained across five independent random seeds using the same fixed train/validation/test split, yielding consistent test-set performance (AUC range: 0.832–0.845; mean ± SD: 0.839 ± 0.005).

#### SBayesR

SBayesR estimates SNP effect weights from the GWAS discovery sample using a Bayesian multiple regression method, enabling the incorporation of approximately one million HapMap3 SNPs into a single PRS score. This approach re-scales GWAS SNP effect estimates based on a mixture of four zero-mean normal distributions, accounting for varying effect sizes across SNPs without requiring a separate tuning cohort [26].

#### Clumping and thresholding (C + T)

The C + T method was applied to construct PRS implemented in PLINK. We applied a linkage disequilibrium (LD) threshold of 0.1 and a *P*-value threshold of 10^− 5^, and an LD window size of 250 kb. SNPs were selected based on these thresholds, and clumps were formed around index SNPs within a defined LD window. A total of 322 SNPs were included using this threshold. The PRS was calculated as the sum of risk alleles weighted by their effect sizes from GWAS summary statistics [27, 28].

### Statistical analysis

Time since study entry was used as the underlying time scale in all survival analyses. A Cox proportional hazards (PH) regression model was used to assess the association of each PRS measure and the time to ADRD onset in a polygenic hazard score (PHS), using the following equation:

$$h\left(t∣X\right)\sim{h}_{0}\left(t\right).{exp}\;(\beta.PRS+\gamma.Age)$$

Where h(t) is the hazard at time t, ${h}_{0}\left(t\right)$ is the baseline hazard, PRS is the polygenic risk scores, and Age is the baseline age. There were no missing values for any of the phenotypic variables among the included individuals. Proportional hazards assumption for Cox models was assessed using Schoenfeld residuals.

Subgroup analyses were conducted to assess stratified effects by *APOE* genotypes and age groups, with age at baseline categorized into seven groups: 40–44, 45–49, 50–54, 55–59, 60–64, 65–69, and 70–74 years. Interaction effects between PRS and *APOE* genotypes were tested using multiplicative interaction terms in generalized linear models (GLMs) and logistic regression, which were used for classification-based analyses (reporting odds ratios), while Cox proportional hazards (PH) models were used separately for time-to-event analyses (reporting hazard ratios). Three-way interactions (PRS × age × *APOE-ε4*) were tested using likelihood ratio tests. Sex was included as a covariate in all models but was not a focus of stratified analysis due to the absence of significant interaction effects. Model performance was evaluated using global discrimination metrics (AUC and C-index) across the full PRS distribution, and stratified comparisons (hazard ratios: HRs, classification accuracy) between the top and bottom PRS quartiles. To assess classification performance, we calculated confusion matrix metrics including sensitivity, specificity, positive predictive value (PPV), and negative predictive value (NPV).

To determine the classification threshold, we used the Youden index, which maximizes the sum of sensitivity and specificity. Additionally, the Area Under the Precision-Recall Curve (AUPRC) was calculated to assess model performance in the context of class imbalance, particularly given the low incidence of ADRD in the cohort. AUPRC was computed using the full test set without subsampling. Multiple comparisons were adjusted using the Bonferroni correction.

#### Software and tools

Various R and Python packages (R version 4.2.2, Python 3.10) were used for statistical analysis and visualization. In R, the survival (v3.8-3) and survminer (v0.5.0) packages were employed for Cox proportional hazards modeling and time-to-event visualization, pROC (v1.18.0) for ROC curve analysis, timeROC (v0.4) for time-dependent ROC analysis, and ggplot2 (v3.4.4) for general data visualization. For constructing the Bayesian variational autoencoders-based PRS (DDML), Python libraries including TensorFlow (v2.12.0), Keras (v2.12.0), and scikit-learn (v1.3.0) were used to implement and evaluate the deep learning models. The SBayesR method was executed using the Genome-wide Complex Trait Bayesian analysis (GCTB) software (v2.0.3), while the clumping and thresholding (C + T) method was applied using PLINK (v1.90b6.21). All PRS values were standardized using the Z-score to ensure comparability across methods. The code used for deep learning analyses and GWAS summary statistics file are publicly available on GitHub (https://github.com/shayanmostafaei/DDML_PRS_ADRD).

#### Data availability

Data from the UK Biobank are available to bona fide researchers upon application (https://www.ukbiobank.ac.uk/enable-your-research/apply-for-access)*.*

## Results

### Participant characteristics

The mean age of the participants was 56.82 (SD = 7.98) years, and 46.69% were men. The median follow-up time was 9.19 (IQR = 1.36) years. Among 276,566 included participants with white British/ European ancestry, 1,328 individuals (0.48%) were diagnosed with ADRD, including AD (37.9%), vascular dementia (16.6%), and mixed or unspecified dementia (45.5%). Of these cases, 14.2% were classified as early-onset and 85.4% as late-onset (Table 1).

**Table 1:** Baseline characteristics of the included UK Biobank participants

Characteristics	All participants (*N* = 276,566)	ADRD (*N* = 1,328)	Non-ADRD (*N* = 275,238)	*P*-value
Age, years (mean ± SD)	56.82 ± 7.98	64.17 ± 5.01	56.79 ± 7.97	< 0.001
*APOE-ε4* carriers (*N*, %)	69,900 (25.27%)	762 (57.38%)	69,138 (25.12%)	< 0.001
Sex, men (*N*, %)	129,131 (46.69%)	750 (56.48%)	128,381 (46.64%)	< 0.001
Follow up time, years (median, IQR)	9.19 (1.36)	6.23 (2.88)	9.19 (1.35)	< 0.001
DDML_PRS, Z-score (mean ± SD)	0.01 ± 1.01	2.70 ± 3.14	0.01 ± 0.90	< 0.001
SBayesR_PRS, Z-score (mean ± SD)	0.01 ± 1.01	2.03 ± 2.05	0.01 ± 0.92	< 0.001
C+T_PRS, Z-score (mean ± SD)	0.01 ± 1.01	1.29 ± 1.69	0.01 ± 0.98	< 0.001

*ADRD:* Alzheimer’s disease and related dementias, *APOE-ε4* carriers: Number and percentage of participants carrying at least one *APOE-ε4* allele, IQR: Interquartile range, PRS values were standardized (Z-scored) using the mean and standard deviation of the full cohort. Mean ± SD values shown for ADRD cases and non-cases therefore reflect subgroup distributions and are not expected to have unit variance.

### Overall predictive performance of PRS models

**Fig. 1:**
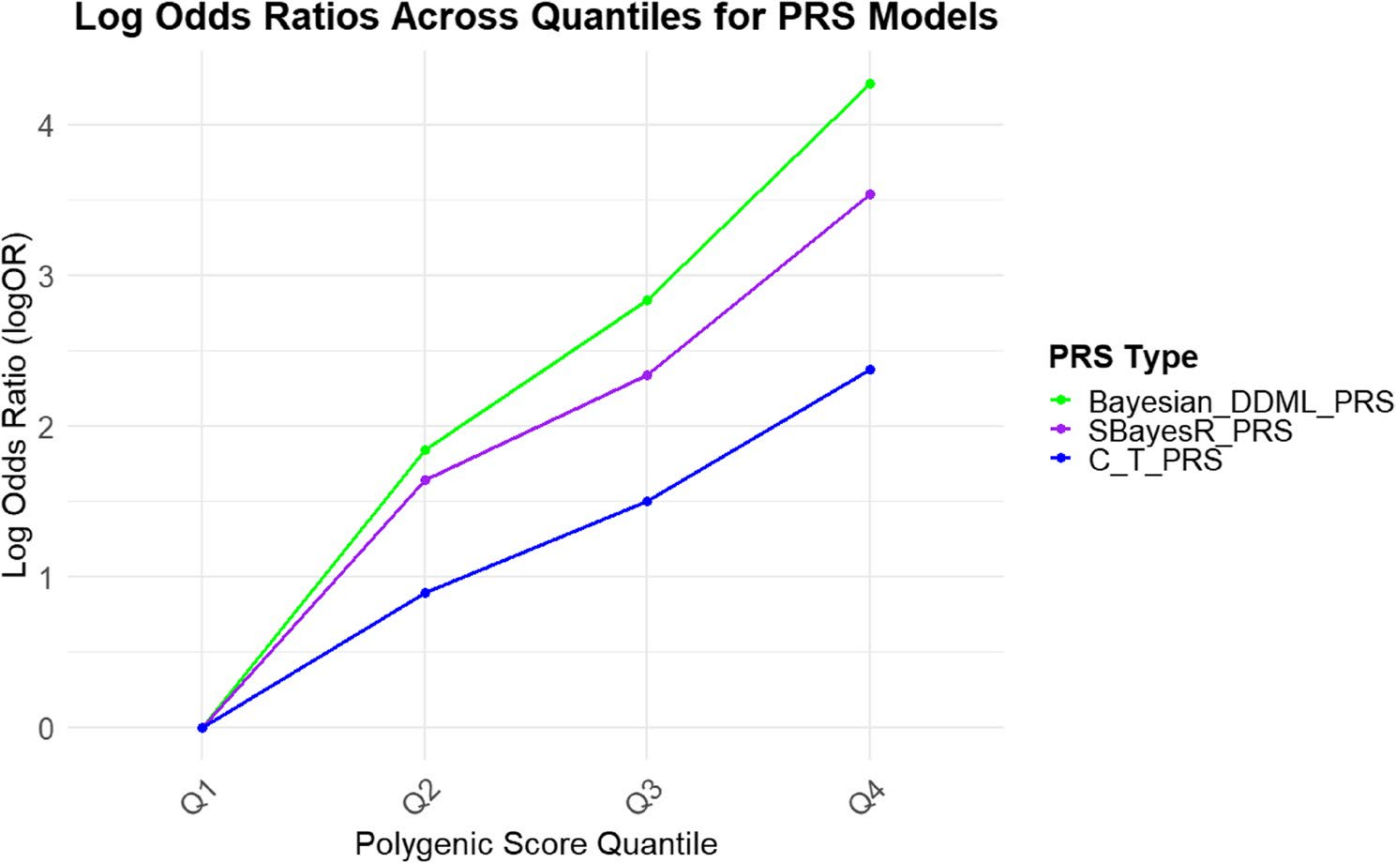
Comparison of log odds ratios (logOR) for Alzheimer’s Disease and Related Dementias (ADRD) across polygenic risk scores (PRS) quartiles for three different PRS models in the UK Biobank (*N* = 276,566). The models include DDML_PRS (green), which integrates deep learning with Bayesian inference; SBayesR_PRS (purple), a summary statistic–based Bayesian regression method; and C + T_PRS (blue), the traditional clumping and thresholding approach. The x-axis represents PRS quartiles (Q1–Q4). The y-axis shows the logOR, indicating the strength of association between genetic risk and ADRD. An increasing trend in logOR from Q1 to Q4 indicates that individuals in higher PRS quantiles have elevated genetic risk for ADRD due to higher genetic risk. DDML_PRS demonstrates the steepest gradient, suggesting superior discrimination of high-risk individuals relative to the other models

**Fig. 2:**
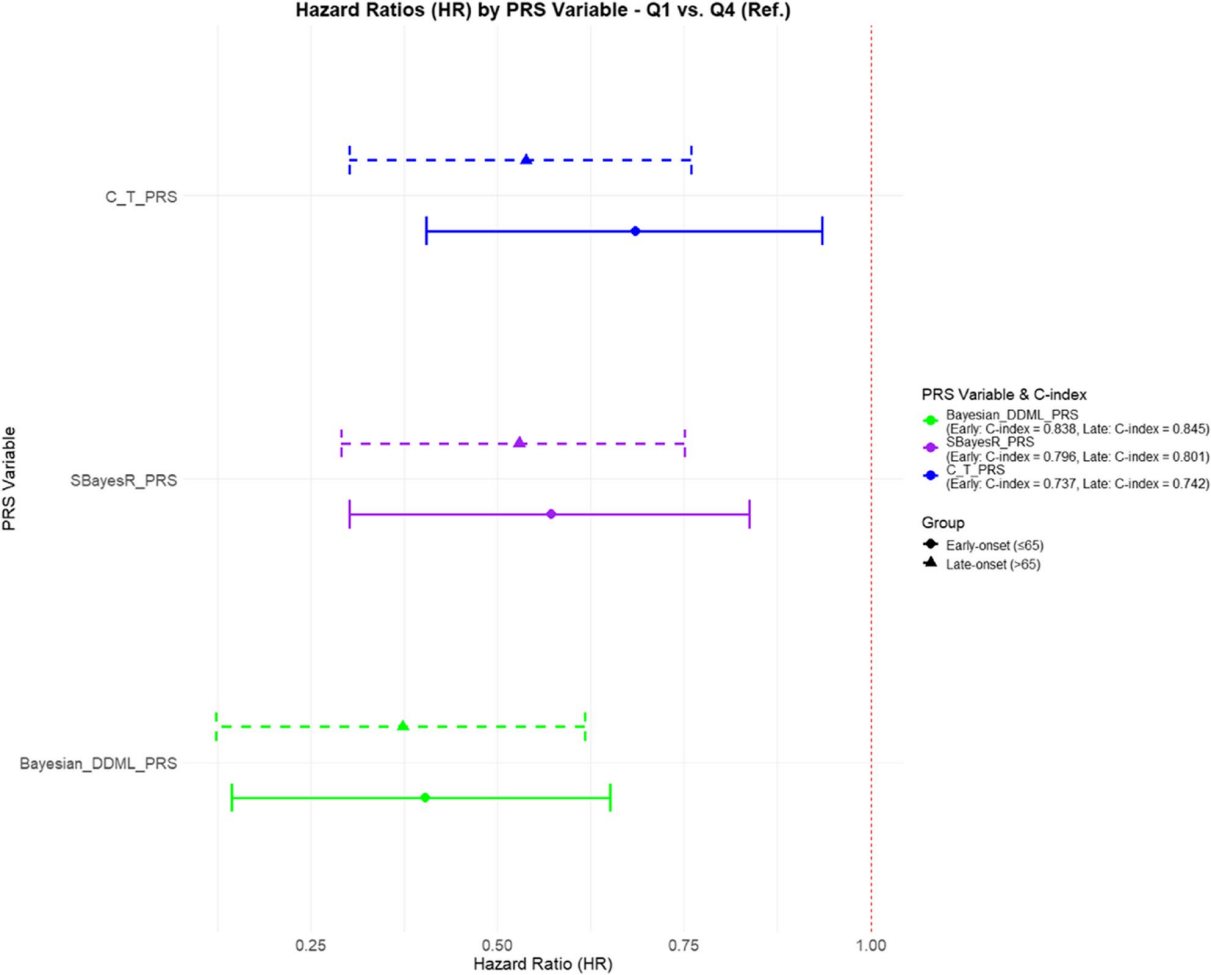
Hazard ratios (HRs) with 95% confidence intervals (CIs) for Alzheimer’s Disease and Related Dementias (ADRD) comparing lowest (Q1) vs. highest (Q4, reference) polygenic risk scores (PRS) quartiles across three PRS models in the UK Biobank cohort (*N* = 276,566). This figure presents HRs and corresponding 95% CIs for ADRD across three PRS models: DDML_PRS (green), SBayesR_PRS (purple), and C + T_PRS (blue). Analyses are stratified by age at onset: early-onset ADRD (onset < 65 years; triangle symbol, dashed line) and late-onset ADRD (onset ≥ 65 years; circle symbol, solid line). For each model, the HR reflects the relative hazard of ADRD in individuals within the lowest PRS quartile (Q1) compared to those in the highest quartile (Q4), used as the reference group (HR = 1). Lower HRs indicate stronger discriminatory power in identifying individuals at reduced genetic risk. C-index values, representing model discrimination performance, are shown in the legend for each PRS and onset group. Among the three models, DDML_PRS shows the highest C-index for both early-onset (0.838) and late-onset (0.845) ADRD, indicating superior predictive accuracy relative to SBayesR_PRS (early: 0.796; late: 0.801) and C + T_PRS (early: 0.737; late: 0.742)

Figure 1 shows the relationship between PRS quartiles and the log odds ratio (LogOR) for ADRD across three different PRS models. Each quartile represents subsets of individuals grouped by their PRS values, with higher quartiles corresponding to greater genetic risk. The LogOR reflects the strength of association between PRS and ADRD, allowing for a comparison of the predictive performance of the models across different risk strata. According to survival models, DDML_PRS (HR = 1.13 per SD [95% CI: 1.13–1.14]), SBayesR_PRS (HR = 1.12 per SD [95% CI: 1.11–1.12]), C + T_PRS (HR = 1.09 per SD [95% CI: 1.09–1.10]) were associated with time to ADRD after adjustment for age at baseline and sex (all *P* < 10^− 5^). These associations remained statistically significant after Bonferroni correction. Using the highest quartile (Q4) as the reference group, the first quartile (Q1) group had around 2-fold lower risk of ADRD for DDML_PRS (Late-onset: HR = 0.37, [95% CI: 0.12–0.62]; Early-onset: HR = 0.53, [95% CI: 0.29–0.75]). In addition, the HRs of Q1 against Q4 for late-onset ADRD were consistently lower than for early-onset ADRD across all PRS models; however, these differences were not statistically significant (Fig. 2).

### Age- and *APOE*-stratified performance

Predictive performance of DDML_PRS, SBayesR_PRS, and C + T_PRS was evaluated in the independent UKB test set using both PRS-only (genetics-only) discrimination and covariate-adjusted models incorporating PRS together with age, sex, and the first 10 genetic principal components, and *APOE* genotype where indicated (Table S1; Table 2). In PRS-only analyses (Table S1), discrimination was modest across all methods, consistent with the low incidence of ADRD in the cohort. When the *APOE* region was included, DDML_PRS achieved the highest PRS-only AUC (0.691, 95% CI: 0.68–0.70), followed by SBayesR_PRS (AUC = 0.660) and C + T_PRS (AUC = 0.612), while a logistic regression benchmark trained on the same 80-SNP panel showed substantially lower discrimination (AUC = 0.594).

**Table 2:** Covariate-adjusted discrimination (AUC) of PRS models in the UK Biobank test set, with and without the *APOE* region included in polygenic risk scores (PRS) construction

PRS model	All participants without *APOE* locus	All participants with *APOE* locus	*P*-value (with vs. without *APOE*)	*APOE-ε4* carriers with model trained on only carriers	*APOE-ε4* non-carriers with model trained on only non-carriers	*P*-value (carriers vs. non-carriers)
DDML_PRS, AUC (95% CI)	0.78 (0.77–0.80)	0.83 (0.83–0.85)	< 0.001	0.84 (0.83–0.85)	0.82 (0.82–0.84)	0.045
SBayesR_PRS, AUC (95% CI)	0.76 (0.75–0.78)	0.79 (0.78–0.80)	< 0.001	0.80 (0.79–0.81)	0.78 (0.77–0.79)	0.021
C + T_PRS, AUC (95% CI)	0.68 (0.67–0.70)	0.73 (0.73–0.75)	< 0.001	0.74 (0.72–0.76)	0.72 (0.71–0.74)	0.040
*P*-value (C + T_PRS as a reference)	*P* < 1 × 10^− 13^	*P* < 1 × 10^− 13^	-	*P* < 1 × 10^− 12^	*P* < 1 × 10^− 12^	-

The area under the receiver operating characteristic curve (AUC) with 95% confidence intervals (CIs) is reported for three PRS models (DDML_PRS, SBayesR_PRS, and C + T_PRS) evaluated in downstream covariate-adjusted logistic regression models including PRS + age + sex + first 10 genetic principal components (PCs). Performance was evaluated (1) in all participants using PRS constructed with and without the *APOE* region, and (2) separately in *APOE-ε4* carriers and non-carriers within the test set, using PRS constructed with the *APOE* region. “Without *APOE* region” indicates exclusion of SNPs within chr19:44–46 Mb (GRCh37/hg19) prior to PRS construction. Statistical comparisons of AUC values were conducted using DeLong’s test to assess the added predictive value of including the *APOE* region and performance differences between *APOE-ε4* carriers and non-carriers for each model. In addition, DDML_PRS showed stable discrimination across datasets: training-set vs. test-set AUC comparisons (performed separately for PRS constructed with and without the *APOE* region) were not statistically significant (all *P*-values > 0.20).

In covariate-adjusted models (Table 2), discrimination improved substantially for all PRS approaches. DDML_PRS showed the highest overall performance (AUC = 0.83, 95% CI: 0.83–0.85 with *APOE*; AUC = 0.78, 95% CI: 0.77–0.80 without *APOE*; *P* < 0.001), outperforming both SBayesR_PRS (AUC = 0.79 with *APOE*) and C + T_PRS (AUC = 0.73 with *APOE*). Stratified analyses further demonstrated higher discrimination among *APOE-ε4* carriers than non-carriers for all methods, with DDML_PRS achieving an AUC of 0.84 (95% CI: 0.83–0.85) in carriers compared with 0.82 (95% CI: 0.82–0.84) in non-carriers (*P* = 0.045). All pairwise AUC comparisons using C + T_PRS as the reference were statistically significant (*P* < 1 × 10⁻¹²).

### Subtype-specific performance across ADRD diagnoses

All PRS models showed the highest predictive performance for AD compared to other ADRD subtypes (Table 3). DDML_PRS showed the highest predictive performance across ADRD subtypes: AD (AUC: 0.86), vascular (AUC: 0.84), and mixed or unspecified dementias (AUC: 0.80). It significantly outperformed SBayesR_PRS and C + T_PRS (*P* < 0.001).

**Table 3:** Performance of PRS models across ADRD subtype subsets in the UK Biobank test set (*APOE* included)

PRS model	AD	Vascular dementia	Other unspecified or mixed dementias	*P*-values (vs. AD)
DDML_PRS, AUC (95% CI)	0.86 (0.85–0.87)	0.84 (0.82–0.85)	0.80 (0.79–0.82)	< 0.05
SBayesR_PRS, AUC (95% CI)	0.82 (0.80–0.83)	0.79 (0.77–0.80)	0.77 (0.76–0.79)	< 0.05
C+T_PRS, AUC (95% CI)	0.76 (0.74–0.78)	0.74 (0.72–0.76)	0.71 (0.70–0.73)	< 0.05
*P*-values (C + T_PRS as a reference)	< 0.001	< 0.001	< 0.001	-

The table reports discrimination performance of the same prespecified DDML_PRS model, trained once on the full training dataset, and evaluated separately within ADRD subtype subsets (Alzheimer’s disease, vascular dementia, and mixed/other dementias) in the independent test set. The last subtype includes cases coded with ICD-10 diagnoses such as F02, F03, G31.0, G31.1, G31.8. No subtype-specific retraining was performed. All models were evaluated using downstream covariate-adjusted regression including PRS, age, sex, the first 10 genetic principal components, and *APOE* genotype. AUCs are reported with 95% confidence intervals.

### Time-dependent predictive performance

**Fig. 3:**
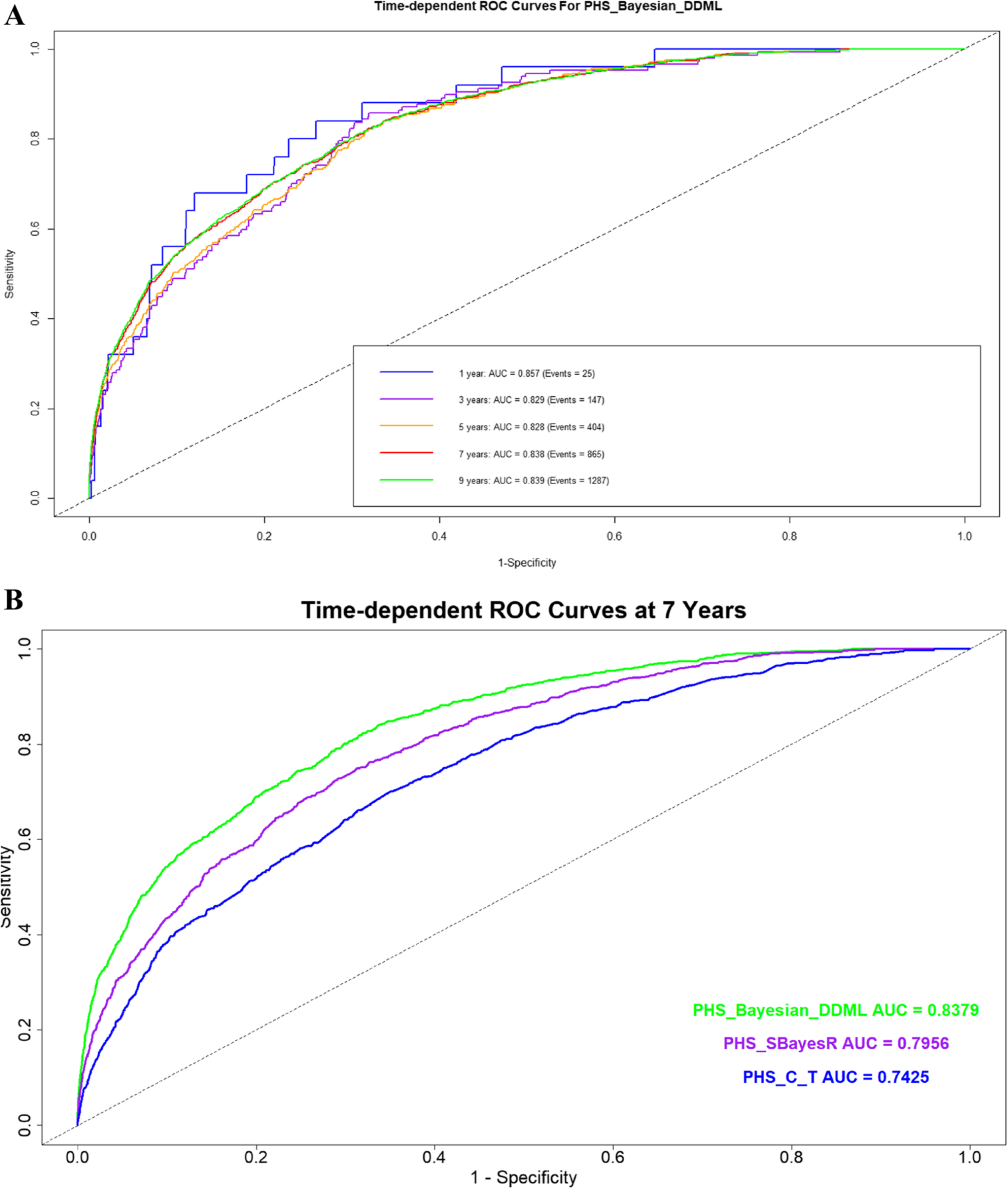
**A**. Time-dependent receiver operating characteristic (ROC) curves for the polygenic hazard scores (PHS) derived from the DDML model for Alzheimer’s Disease and Related Dementias (ADRD) in the UK Biobank cohort (*N* = 276,566). This figure shows the temporal performance of the DDML-based PHS in predicting ADRD risk over follow-up time. Time-dependent ROC curves were computed at multiple time points to assess the model’s ability to distinguish between individuals who developed ADRD and those who did not. The area under the ROC curve (AUC) at each time point quantifies the model’s discriminatory accuracy, with higher AUC values indicating better predictive performance. This analysis demonstrates the DDML_PHS model’s robustness in long-term risk prediction. **B**. Receiver operating characteristic (ROC) curves for all polygenic hazard scores (PHS) models at 7 years of follow-up for Alzheimer’s Disease and Related Dementias (ADRD) in the UK Biobank testing set (*N* = 92,188). This figure compares the classification performance of PHS models: PHS_DDML (green), PHS_SBayesR (purple), and PHS_C + T (blue), based on their ability to predict incident ADRD cases over a 7-year follow-up period. ROC curves were generated using the testing dataset, and the area under the ROC curve (AUC) was calculated for each model. Statistical comparisons of AUCs using DeLong’s test indicated that PHS_DDML significantly outperformed PHS_SBayesR, with PHS_C + T serving as the reference model

The AUC for each PHS model was calculated at 1 year, 3 years, 5 years, 7 years, and 9 years of follow-up. While the AUC at 1 year was the highest (AUC = 0.86), it was based on a limited number of ADRD events (*n* = 25), making this estimate less reliable. Predictive performance remained consistently strong from 5 years onward, with AUCs stabilizing around 0.83–0.84 and was supported by a larger number of observed ADRD events (Fig. 3A). At the 7-year follow-up, PHS_DDML demonstrated the highest discriminative ability (AUC = 0.84), followed by PHS_SBayesR (AUC = 0.80) and PHS_C + T (AUC = 0.74) (Fig. 3B).

### Interaction effects between prs, age, and *APOE*

The interactions between PRSs (excluding the *APOE* region) and *APOE-ε4* carrier status were statistically significant, with the strongest interaction observed for DDML_PRS (*β* = 0.16, 95% CI: 0.12–0.21, *P* = 3 × 10⁻^15^). Similarly, statistically significant interactions were found between PRSs and age, with DDML_PRS again showing the strongest interaction (*β* = 0.016, 95% CI: 0.014–0.018, *P* < 2 × 10⁻^16^) using logistic regression. The performance of each PRS across different age strata further demonstrated interaction effects between age and PRSs (Supplementary Fig. 2). Our analysis indicates that *APOE-ε4* status statistically significantly increases PRS model performance in predicting ADRD, particularly in older age groups. The DDML_PRS model showed improved AUC in *APOE-ε4* carriers, especially in individuals aged 70 and above. Similarly, SBayesR_PRS and C + T_PRS models also demonstrated higher AUCs in *APOE-ε4* carriers, with SBayesR_PRS showing the greatest advantage in the 70 + age group (Supplementary Figure 3). Statistical tests for the interaction effects of PRSs-by-age-by-*APOE-ε4* carrier status, showed statistically significant three-way interactions for all PRSs (likelihood ratio test *P* < 0.05). The predictive power of PRS models was slightly higher for *APOE-ε4* carriers in the AD subtype of ADRD (Supplementary Figure 4). Additionally, the PRS models showed slightly higher predictive power in women compared to men across all PRSs (e.g., DDML_PRS: AUC = 0.84 in women vs. 0.84 in men; *P* = 0.46), indicating no significant sex-based difference.

### Classification accuracy and model comparisons

Based on the confusion matrix (calculated using the optimal sensitivity-to-specificity threshold derived via the Youden index), DDML_PRS model achieved classification accuracy ranging from 63.97% to 66.61% across age groups at baseline, with classification performance generally improving with increasing age (Table S2). The optimal threshold for DDML_PRS was 0.1461, yielding an AUC of 0.839, sensitivity of 0.786, specificity of 0.718, balanced accuracy of 0.752, PPV of 0.013, and NPV of 0.999 in the whole cohort. These metrics illustrate the model’s strong discriminative ability despite modest apparent accuracy, which is expected given the extremely low prevalence of ADRD. In terms of the AUPRC, DDML_PRS achieved a value of 0.077 on the full independent test set, computed without subsampling or averaging. Given the low ADRD prevalence, this value reflects the model’s ranking ability rather than absolute classification accuracy.

Specifically, the proportion of correctly classified individuals increased from 63.97% in the 45–49 age group to 66.61% in the 70–74 age group. The Cochran-Armitage trend test confirmed a statistically significant increasing trend in classification accuracy (CC) across age groups (*Z-value* = 3.61, *P* < 0.0003). To contextualize the performance of the DDML_PRS model, we compared it with a simpler age-only model using the same classification threshold. Across all age groups, the PRS-informed model (PRS + age) achieved a modest but consistent increase in the number of correctly classified individuals compared with the age-only model, reflecting improved sensitivity to ADRD cases while maintaining high specificity. The largest absolute gains in correct classification were observed in older age groups, with increases of 308 and 633 correctly classified individuals in the 60–64 and 65–69 age groups, respectively (Table S3).

We also evaluated the model’s performance among *APOE-ε4* carriers vs. non-carriers with *APOE* region (Table S4). Although the model performed similarly across both groups, a statistically significant but modest improvement in classification accuracy was observed among carriers in the 65–69 age group (63.09% vs. 62.82%; *P* = 0.048). No significant differences were found in other age groups. Furthermore, no differences were found for DDML_PRS model performances in men and women across age groups (Table S5).

Across all stratified analyses, DDML_PRS consistently demonstrated superior predictive performance. Notably, AUCs were highest in *APOE-ε4* carriers (0.84), in AD subtype cases (0.86), and in older age groups (up to 66.61% classification accuracy in ages 70–74).

## Discussion

To our knowledge, this is the first study to apply a deep learning–based PRS model (DDML_PRS) using Bayesian variational autoencoders for ADRD risk prediction in a large population-based cohort. In this large cohort study, all the PRS models (DDML_PRS, SBayesR_PRS, C + T_PRS) were significantly associated with ADRD risk. In covariate-adjusted models, DDML_PRS achieved the highest discrimination (AUC = 0.83 when the *APOE* region was included), consistently outperforming the comparator PRS methods. In contrast, PRS-only (genetics-only) discrimination was modest across all methods, including DDML_PRS, highlighting the limited standalone predictive value of common genetic variation in this setting and the dominant contribution of established demographic and genetic risk factors. Compared with a standard logistic regression benchmark trained on the same 80 SNPs (PRS-only AUC ≈ 0.60), DDML_PRS demonstrated higher discrimination, suggesting that its performance reflects the integration of a constrained Bayesian architecture with downstream covariate modeling rather than reliance on *APOE* or cohort-specific effects alone. Importantly, both age and *APOE* genotype emerged as strong modifiers of PRS performance, with higher discrimination observed in older individuals and *APOE-ε4* carriers. While the peak AUCs observed in covariate-adjusted models approach ~ 0.85 in specific age strata, these values should be interpreted cautiously, as they reflect the combined contribution of genetic risk scores with age and *APOE* rather than genetics alone, and may be influenced by cohort characteristics and limited disease incidence within UKB. Several factors may explain this discrepancy. First, our DDML_PRS model incorporates a Bayesian variational autoencoder architecture that captures complex, higher-order genetic representations among SNPs, which are not modeled in traditional linear PRS approaches. Second, our cohort was restricted to unrelated individuals of European ancestry with stringent quality control and inclusion of the first 10 genetic PCs, which may reduce heterogeneity and improve signal-to-noise ratio. Third, we evaluated model performance using a strict holdout validation strategy and included known predictors such as age, sex, and *APOE* genotype, which substantially improved discrimination. Notably, Thompson et al. [17] report AUC estimates around 0.69 for AD prediction in UKB using their PRS release [17]. Importantly, PRS comparisons in the literature may differ in whether the *APOE* region is retained within the PRS versus removed and modelled separately via *APOE* ε2/ε3/ε4 genotype. To avoid conflating these concepts, we explicitly distinguish “with *APOE* region” versus “without *APOE* region” PRS construction, and we report PRS-only versus covariate-adjusted performance separately. Our C + T PRS achieved an AUC of 0.68, closely matching Thompson’s result, further validating the comparability of our baseline model. When these covariates are incorporated, DDML_PRS AUC estimates (~ 0.83–0.86 over follow-up time) align more closely with upper bounds reported in biomarker-confirmed cohorts [29]. To improve transparency, we have also reported DDML_PRS performance under different covariate specifications (e.g., PRS-only, PRS+*APOE*, PRS+*APOE* + age+sex), which further contextualizes our results. Nevertheless, we acknowledge that performance estimates may be cohort-specific and potentially inflated due to sample characteristics. Future validation in external cohorts, particularly those with biomarker-confirmed diagnoses and diverse ancestries, is essential to confirm generalizability and clinical utility. While our analysis was restricted to individuals of European ancestry, we acknowledge that this limits the generalizability of our findings. This design choice was made to reduce population stratification and ensure robust evaluation of the DDML_PRS architecture in a genetically homogeneous cohort. Evaluating cross-ancestry performance is an important next step, but was beyond the scope of this study, which focused on testing and benchmarking the model.

DDML_PRS model showed improved classification accuracy in *APOE-ε4* carriers, particularly among individuals aged 65 years and older (*P* = 0.048). This likely reflects the model’s sensitivity to polygenic architecture typical of late-onset ADRD, where age and *APOE* genotype play a more pronounced role. Subgroup analyses across ADRD subtypes indicated consistent trends, although predictive strength varied slightly by diagnosis category. The lower AUCs observed for vascular and mixed dementias may reflect greater clinical and etiological heterogeneity, as well as limitations in ICD-10 coding accuracy for these subtypes. These conditions may involve non-genetic risk factors or mixed pathologies that are less well captured by PRS models trained on AD-specific GWAS data. Moreover, diagnostic variability and overlap in clinical presentation can introduce misclassification, further attenuating genetic signal detection. These limitations highlight the need for cautious interpretation of subtype-specific results. No significant three-way interaction was found for PRS, age, and sex. The absence of significant sex-specific effects in our model may also reflect cohort-specific factors (e.g., age) and selection bias in the UKB cohort, which tends to underrepresent older and clinically affected individuals. This demographic skew may obscure true sex differences in ADRD risk, and future studies in more representative cohorts are needed to validate these findings. Together, these findings underscore the potential of polygenic models, particularly those incorporating *APOE* and age-specific effects, for enhancing individualized ADRD risk prediction in clinical and research settings. However, we note that while AUC values were high, classification accuracy was modest (~ 64–66%) due to the low prevalence of ADRD in the cohort. This discrepancy highlights the limitations of threshold-dependent metrics such as accuracy and PPV in rare disease contexts. For example, classifying all individuals as controls would yield an accuracy of ~ 99.5% but zero sensitivity, while classifying all as cases would yield 100% sensitivity but only 0.5% accuracy. Therefore, threshold-independent metrics like AUC and AUPRC provide a more reliable assessment of model performance in imbalanced datasets.

Compared to other genetics-based methods, our proposed Bayesian variational autoencoder -based PRS method (DDML_PRS) achieved high predictive accuracy for ADRD in the UKB when the *APOE* region was included (AUC = 0.83, 95% CI: 0.83–0.85), outperforming traditional models such as C + T and SBayesR and performing comparably to recent state-of-the-art approaches [25, 30]. Notably, this level of accuracy was obtained using only genetic data and a curated subset of known ADRD-associated SNPs [9]. While the model demonstrates strong relative performance among genetic models, it is important to note that a substantial proportion of cases (~ 35%) remain undetected, indicating limitations in clinical applicability. Clinical implementation will require prospective validation, integration with non-genetic risk factors, and demonstration of utility in diverse healthcare settings. Predictive performance was slightly higher in women and improved with age, with the strongest classification observed in individuals aged 65–74. These findings highlight the value of integrating genetic risk, particularly in age- and *APOE*-stratified contexts, to enhance individualized ADRD risk prediction [31].

Our findings are consistent with prior studies showing that meaningful discrimination of ADRD risk is achieved primarily when PRS are combined with established covariates such as age and *APOE* genotype. In this large population-based cohort, all PRS methods were significantly associated with ADRD risk, with DDML_PRS demonstrating the highest predictive accuracy in covariate-adjusted models (AUC up to 0.83 when including the *APOE* region). This level of performance is comparable to previous reports integrating PRS with demographic and genetic risk factors, including Li et al. [32] and Escott-Price et al. [33], and exceeds that of several recent studies using conventional PRS approaches alone. In contrast, PRS-only models in our study showed modest discrimination across all methods, consistent with the low incidence of ADRD in UKB (Table S6). This performance advantage may stem from the model’s ability to capture complex, higher-order genetic representations and incorporate prior GWAS knowledge through Bayesian inference.

Our study found that the AUC of our models increased with age. However, this trend was partially obscured by the large confidence intervals in the younger age groups, likely due to the smaller sample sizes and fewer events in these groups. We hypothesize that this trend may also reflect underlying biological aging differences, as early-onset and late-onset ADRD are genetically distinct, with early-onset cases more likely to involve monogenic causes. Additionally, varying follow-up durations across age groups may have contributed. While the overall median follow-up time was 9.19 years, participants who developed ADRD had a shorter follow-up (median: 6.23 years), potentially limiting the ability to capture long-term predictive performance in some subgroups. Although our Cox models used time since baseline as the time scale and adjusted for age, we acknowledge that informative censoring due to shorter follow-up among ADRD cases may still influence hazard estimates. Since the annual incidence rate of late-onset AD increases with age [34], older individuals had a higher likelihood of receiving an AD diagnosis within the follow-up period. Importantly, we cannot determine whether individuals who did not develop AD during the follow-up might have been diagnosed later. For example, if someone aged 40–44 is correctly predicted to develop AD but is diagnosed after follow-up, the model would falsely appear inaccurate (Table S7). This limitation applies across all age groups, as even individuals aged 75 may still be too young to manifest ADRD. Such scenarios are more common in younger age groups due to their lower AD incidence rates, placing constraints on the AUC in these groups. This could also explain why our model underperformed compared to using age alone as a predictor for individuals aged 40–54. These findings indicate that the AUCs of the second-oldest age group (65–69, years) most accurately represent our model performance and should be used for benchmarking, as the small sample size in the oldest age group (70–74, years) results in too wide confidence intervals.

Our study also found that the predictive capacity of our models was higher among *APOE-ε4* carriers compared to non-carriers aged 65 years and older, a pattern previously described by Escott-Price et al. [35]. They attributed this to survival bias, as *APOE-ε4* carriers are less prevalent in older populations. Since *APOE-ε4* is a known risk factor for developing AD at an earlier age [36], carriers are more likely to be diagnosed within the follow-up period, which may contribute to the stronger model performance in this group. Consequently, fewer individuals in the 70–74 age group are likely to carry this allele due to earlier onset and potential survival bias. Additionally, the stronger performance in *APOE-ε4* carriers may reflect higher genetic penetrance and earlier onset, which increases the likelihood of diagnosis during follow-up. This may inflate model performance in this subgroup and warrants cautious interpretation when generalizing to broader populations. Given that *APOE-ε4* is an important predictor of AD, it is unsurprising that our models perform less effectively in individuals without this key predictor. This discrepancy may explain the stronger predictive performance observed among *APOE-ε4* carriers in this age group.

Our DDML_PRS method demonstrates impressive predictive accuracy for predicting ADRD using genetic data. However, it is important to compare this approach with other emerging diagnostic tools for Alzheimer’s disease. Recent studies highlight the potential of blood tests to identify Alzheimer’s disease with remarkable accuracy. Our method achieves an AUC of 0.83 (95% CI: 0.83–0.85) and an accuracy ranging from 63.97% to 66.61%, depending on the age group. In comparison, the APS2 blood-based biomarker method, which combines plasma amyloid-β 42:40 ratios with plasma p-tau217, achieves an accuracy of 90% in diagnosing clinical Alzheimer’s disease [37]. However, the APS2 method is limited to patients already exhibiting cognitive decline, whereas our genetic-based approach was done in the general population and can be applied at any stage of life, as genetic information is static. Additionally, our results can be compared with an AI-guided predictive model that integrates cognitive tests and structural MRI data in patients with mild cognitive impairment [38]. While our method shows slightly lower predictive performance compared to blood-based or MRI-based tests, it offers important advantages by relying solely on genetic information. This simplifies data collection and enables risk assessment earlier in life, before symptoms or detectable biomarker changes appear. Genetic predictions are particularly valuable because they remain stable over time, potentially reducing the need for invasive and repeated testing. Moreover, they provide insights into the genetic architecture of ADRD, potentially guiding the discovery of novel therapeutic targets. Nonetheless, PRS-only approaches have inherent limitations. Unlike biomarker-based methods, which reflect current pathophysiological changes, PRS models capture static genetic risk and cannot detect disease progression or onset. Therefore, while useful for early-life risk stratification, PRS models should be viewed as complementary tools rather than standalone diagnostic solutions. Importantly, genetic risk scores could also be integrated with other biomarkers to improve predictive models, supporting a more precise and personalized approach to ADRD prevention and diagnosis.

The model’s reproducibility across five independent runs and its stable performance metrics are detailed (Table S8), supporting the reliability of the DDML_PRS framework. Traditional PRS methods, including those based on clumping and thresholding (C + T), face limitations such as potential bias in variant selection and failure to accurately reflect the real effect size of correlated variants. Moreover, they often fail to capture non-linear effects and interactions between genetic variants. SBayesR, which is an improvement over traditional methods, still depends on linear (additive) regression and may not fully capture the complex, non-linear or higher-order relationships among genetic variants [26]. In contrast, our PRS approach (DDML_PRS) offers several advantages: it can encapsulate variant interactions and non-linear effects with fewer presumptions about genetic architecture, adapt to different genetic architectures more robustly across phenotypes (ADRD), and better handle challenges of overfitting and potential overestimation of effect sizes in correlated variants [25]. The novelty of our DDML_PRS approach lies in its ability to capture complex, non-linear relationships between genetic variants, potentially uncovering intricate patterns that traditional PRS methods might miss due to their reliance on weighted linear functions of associated SNPs [39, 40]. This method combines the strengths of variational autoencoders with Bayesian inference, incorporating prior information from GWAS summary statistics [23]. This integration allows for a more robust and uncertainty-aware model. In the context of PRS construction, DDML can be applied to learn latent representations of genetic data that capture complex SNP-SNP interactions and non-linear relationships between genetic variants and ADRD risk, potentially offering insight into which variants contribute most and how they interact in ADRD risk prediction [41–43]. Despite these advantages, our genetic model provides valuable insight into ADRD risk, but its predictive power is limited by the complex, multifactorial nature of the disease. With an accuracy of 63.7%–66.61%, many at-risk individuals may go undetected, limiting clinical utility. The low ADRD prevalence in the UKB (~ 0.48%), likely due to younger baseline age, may further impair model performance due to case underreporting. Validation studies on the UK Biobank showed UKB dementia data is highly accurate when recorded (PPV ~ 82.5%), but even with linked registries, 14–79% of true cases may be missed [14, 44]. This level of under-detection and diagnostic uncertainty may lead to misclassification bias, which would likely attenuate observed associations and reduce statistical power. Consequently, PRS-ADRD effect sizes may be underestimated, and the model’s ability to detect true genetic signals may be impaired. Future studies should validate PRS models using biomarker-confirmed diagnoses or clinically adjudicated outcomes to improve phenotype precision and enhance the reliability of genetic risk estimates. External replication was not undertaken in the present study due to practical constraints, including differences in available genotyping content and phenotype definitions across cohorts and limited sample sizes for performing comparable age- and *APOE*-stratified analyses. Additionally, the higher ADRD prevalence in men within UKB contrasts with global trends and could skew PRS performance if sex-specific genetic effects are not explicitly modeled. Importantly, the study population consisted exclusively of individuals of European/white ancestry, which limits the generalizability of our findings to other ethnic groups. Future validation in more diverse populations is essential to evaluate the transferability and equity of PRS-based risk prediction. Participation in UKB may introduce selection bias, as both *APOE* genotype and age influence participation and ADRD risk. Conditioning or stratifying on these factors can induce collider bias (e.g., *APOE* → participation → AD), potentially distorting associations. While our models adjusted for age and *APOE*, we observed that excluding *APOE* from training modestly reduced performance (DDML_PRS: AUC = 0.78 [0.77–0.80] vs. 0.83 [0.83–0.85] when included), indicating both *APOE*’s strong contribution and the residual predictive value of genome-wide polygenic signal. To mitigate bias, future work should formally assess and correct for selection mechanisms, for instance, through inverse probability weighting (IPW) informed by causal directed acyclic graph (DAG) analysis. Additionally, methods such as Mendelian randomization may help disentangle causality from correlation in genetic datasets, and machine learning techniques capable of modeling complex interactions without conditioning on colliders could further reduce bias and improve predictive accuracy. We also acknowledge that the proportions of ADRD subtypes in our cohort, with AD accounting for 37.9%, vascular dementia for 16.6%, and mixed/unspecified dementia for 45.5%, differ from population-based estimates. Therefore, subtype-specific performance metrics should be interpreted with caution.

Importantly, genetic and non-genetic approaches are not mutually exclusive. As discussed above, the ideal future of ADRD assessment may involve a combination of genetic risk assessment, blood-based biomarker tests, and clinical evaluation including cognition testing to provide a comprehensive and accurate prediction. These findings support a multi-modal strategy, showing that DDML_PRS achieved the highest predictive accuracy (AUC = 0.84), particularly in *APOE-ε4* carriers and older adults, and significantly outperformed simpler models. This complementary approach leverages the strengths of each modality, potentially improving early detection, treatment efficacy, and patient outcomes. Our results support the development of integrated risk prediction frameworks that combine genetic, biomarker, and clinical data to enable earlier, more accurate, and personalized dementia prevention strategies.

## Supplementary Information

Supplementary Material 1. Supplementary Figure 1. Flow chart for selecting UK Biobank (UKB) participants. Participants were selected based on self-identified ancestry using Data-Field 22006. A total of 339,332 individuals without kinship were identified using Data-Field 22021.

Supplementary Material 2. Supplementary Figure 2. Area under the ROC curve (AUC) for polygenic risk scores (PRSs) across age groups in the UK Biobank. AUC values for all PRSs were evaluated on the testing set (*N* = 92,188), stratified by age at baseline in 5-year intervals.

Supplementary Material 3. Supplementary Figure 3. AUC for PRSs stratified by *APOE-ε4* carrier status and age in the UK Biobank. Performance of all PRSs on the testing set (*N* = 92,188), stratified by *APOE-ε4* carrier status and age at baseline.

Supplementary Material 4. Supplementary Figure 4. AUC for PRS models by *APOE-ε4* status and ADRD subtypes in the UK Biobank. AUC values for all PRS models on the testing set (*N* = 92,188), stratified by *APOE-ε4* carrier status and Alzheimer’s Disease and Related Dementias (ADRD) subtypes. Subtypes include Alzheimer’s Disease (AD), vascular dementia, and other, unspecified, or mixed dementias.

Supplementary Material 5. Supplementary Table 1. PRS-only (no covariates) discrimination for ADRD in the UK Biobank with and without the APOE region. Supplementary Table 2. Confusion matrix metrics (N) for the top performing PRS (i.e., DDML_PRS) across age groups at baseline on the whole sample (*N*= 276,566). Supplementary Table 3. The number of correctly classified individuals by the top performing PRS (i.e., DDML_PRS) in the UK Biobank (*N*=276,566) with prevalence of 0.48%. Supplementary Table 4. Comparison between the number (%) of correctly classified individuals (CC) by the top performing model (i.e., DDML_PRS) among APOE-ε4 carriers and non-carriers in the UK Biobank (*N*=276,566) and prevalence of 0.48% . Supplementary Table 5. The number of correctly classified individuals (CC) by the top performing PRS (i.e., DDML_PRS) between women and men in the UK Biobank (*N*=276,566) and prevalence of 0.48%. Supplementary Table 6. Comparison of predictive accuracy of PRS (AUC, 95% CI) for any dementia type or mild-cognitive impairment (MCI) prediction from this study and published studies [32, 33, 45–56]. Supplementary Table 7. Population summary of age at baseline, ADRD cases, follow-up period in ADRD cases, and age at diagnosis in the UK Biobank (*N* = 276,566, prevalence of ADRD = 0.48%). Supplementary Table 8. Model specification details for the DDML_PRS framework used in ADRD risk prediction.

## Data Availability

Data from the UK Biobank are available to bona fide researchers upon application (https://www.ukbiobank.ac.uk/enable-your-research/apply-for-access).

## References

[1] Better MA. Alzheimer’s disease facts and figures. Alzheimers Dement. 2023;19(4):1598–695. 36918389 10.1002/alz.13016

[2] Mehta RI, Schneider JA. What is ‘Alzheimer’s disease’? The neuropathological heterogeneity of clinically defined Alzheimer’s dementia. Curr Opin Neurol. 2021;34(2):237–45. 33591030 10.1097/WCO.0000000000000912

[3] Li X, Feng X, Sun X, Hou N, Han F, Liu Y. Global, regional, and national burden of Alzheimer’s disease and other dementias, 1990–2019. Front Aging Neurosci. 2022;14:937486. 36299608 10.3389/fnagi.2022.937486 PMC9588915

[4] Nichols E, Steinmetz JD, Vollset SE, Fukutaki K, Chalek J, Abd-Allah F, et al. Estimation of the global prevalence of dementia in 2019 and forecasted prevalence in 2050: an analysis for the Global Burden of Disease Study 2019. Lancet Public Health. 2022;7(2):e105–25. 34998485 10.1016/S2468-2667(21)00249-8 PMC8810394

[5] Carmona S, Hardy J, Guerreiro R. The genetic landscape of Alzheimer disease. Handb Clin Neurol. 2018;148:395–408. 29478590 10.1016/B978-0-444-64076-5.00026-0

[6] Liu C-C, Kanekiyo T, Xu H, Bu G. Apolipoprotein E and Alzheimer disease: risk, mechanisms and therapy. Nat Reviews Neurol. 2013;9(2):106–18. 10.1038/nrneurol.2012.263 PMC3726719 23296339

[7] Neu SC, Pa J, Kukull W, Beekly D, Kuzma A, Gangadharan P, et al. Apolipoprotein E genotype and sex risk factors for Alzheimer disease: a meta-analysis. JAMA Neurol. 2017;74(10):1178–89. 28846757 10.1001/jamaneurol.2017.2188 PMC5759346

[8] Eissman JM, Wells G, Khan OA, Liu D, Petyuk VA, Gifford KA, et al. editors. Polygenic resilience score may be sensitive to preclinical Alzheimer’s disease changes. Pacific symposium on biocomputing 2023: Kohala Coast, Hawaii, USA, 3–7 January 2023; World Scientific. 2022. PMC9888419 36540999

[9] Bellenguez C, Küçükali F, Jansen IE, Kleineidam L, Moreno-Grau S, Amin N, et al. New insights into the genetic etiology of Alzheimer’s disease and related dementias. Nat Genet. 2022;54(4):412–36. 35379992 10.1038/s41588-022-01024-z PMC9005347

[10] Kunkle B, Grenier-Boley B, Sims R, Bis J, Damotte V, Naj A, et al. Genetic and Environmental Risk in AD/Defining Genetic, Polygenic and Environmental Risk for Alzheimer’s Disease Consortium (GERAD/PERADES). Genetic meta-analysis of diagnosed Alzheimer’s disease identifies new risk loci and implicates Aβ, tau, immunity and lipid processing. Nat Genet. 2019;51(3):414–30. 30820047 10.1038/s41588-019-0358-2 PMC6463297

[11] Carrasquillo MM, Crook JE, Pedraza O, Thomas CS, Pankratz VS, Allen M, et al. Late-onset Alzheimer’s risk variants in memory decline, incident mild cognitive impairment, and Alzheimer’s disease. Neurobiol Aging. 2015;36(1):60–7. 25189118 10.1016/j.neurobiolaging.2014.07.042 PMC4268433

[12] Sudlow C, Gallacher J, Allen N, Beral V, Burton P, Danesh J, et al. UK biobank: an open access resource for identifying the causes of a wide range of complex diseases of middle and old age. PLoS Med. 2015;12(3):e1001779. 25826379 10.1371/journal.pmed.1001779 PMC4380465

[13] Bycroft C, Freeman C, Petkova D, Band G, Elliott LT, Sharp K, et al. The UK Biobank resource with deep phenotyping and genomic data. Nature. 2018;562(7726):203–9. 30305743 10.1038/s41586-018-0579-z PMC6786975

[14] Wilkinson T, Schnier C, Bush K, Rannikmäe K, Henshall DE, Lerpiniere C, et al. Identifying dementia outcomes in UK Biobank: a validation study of primary care, hospital admissions and mortality data. Eur J Epidemiol. 2019;34:557–65. 30806901 10.1007/s10654-019-00499-1 PMC6497624

[15] Gouveia C, Gibbons E, Dehghani N, Eapen J, Guerreiro R, Bras J. Genome-wide association of polygenic risk extremes for Alzheimer’s disease in the UK Biobank. Sci Rep. 2022;12(1):8404. 35589863 10.1038/s41598-022-12391-2 PMC9120074

[16] Leonenko G, Baker E, Stevenson-Hoare J, Sierksma A, Fiers M, Williams J, et al. Identifying individuals with high risk of Alzheimer’s disease using polygenic risk scores. Nat Commun. 2021;12(1):4506. 34301930 10.1038/s41467-021-24082-z PMC8302739

[17] Thompson DJ, Wells D, Selzam S, Peneva I, Moore R, Sharp K, et al. A systematic evaluation of the performance and properties of the UK Biobank Polygenic Risk Score (PRS) Release. PLoS ONE. 2024;19(9):e0307270. 39292644 10.1371/journal.pone.0307270 PMC11410272

[18] Xu X, Li Y, Wang J, Cao Y, Feng C, Guo Y, et al. Family History of AD/Dementia, Polygenic Risk Score for AD, and Parkinson’s Disease. Mov Disorders Clin Pract. 2023;10(12):1787–94. 10.1002/mdc3.13919 PMC10715357 38094649

[19] Gao XR, Chiariglione M, Qin K, Nuytemans K, Scharre DW, Li Y-J, et al. Explainable machine learning aggregates polygenic risk scores and electronic health records for Alzheimer’s disease prediction. Sci Rep. 2023;13(1):450. 36624143 10.1038/s41598-023-27551-1 PMC9829871

[20] Mak JK, McMurran CE, Hägg S. Clinical biomarker-based biological ageing and future risk of neurological disorders in the UK Biobank. J Neurol Neurosurg Psychiatry. 2024;95(5):481–4. 37926442 10.1136/jnnp-2023-331917 PMC11041565

[21] Jansen IE, Savage JE, Watanabe K, Bryois J, Williams DM, Steinberg S, et al. Genome-wide meta-analysis identifies new loci and functional pathways influencing Alzheimer’s disease risk. Nat Genet. 2019;51(3):404–13. 30617256 10.1038/s41588-018-0311-9 PMC6836675

[22] Li X, Kharitonova E, Pang M, Wen J, Zhou LY, Raffield L et al. Variational autoencoder-based model improves polygenic prediction in blood cell traits. Hum Genet Genomics Adv. 2025;6(4):100490. 10.1016/j.xhgg.2025.100490 PMC12398231 40783786

[23] Zabad S, Gravel S, Li Y. Fast and accurate Bayesian polygenic risk modeling with variational inference. Am J Hum Genet. 2023;110(5):741–61. 37030289 10.1016/j.ajhg.2023.03.009 PMC10183379

[24] Vivek S, Faul J, Thyagarajan B, Guan W. Explainable variational autoencoder (E-VAE) model using genome-wide SNPs to predict dementia. J Biomed Inform. 2023;148:104536. 37926392 10.1016/j.jbi.2023.104536 PMC11106718

[25] Li X, Pang M, Wen J, Zhou LY, Raffield LM, Zhou H et al. Variational Autoencoder-based Model Improves Polygenic Prediction in Blood Cell Traits. bioRxiv. 2025;2025(01):13.632820. 10.1016/j.xhgg.2025.100490 PMC12398231 40783786

[26] Lloyd-Jones LR, Zeng J, Sidorenko J, Yengo L, Moser G, Kemper KE, et al. Improved polygenic prediction by Bayesian multiple regression on summary statistics. Nat Commun. 2019;10(1):5086. 31704910 10.1038/s41467-019-12653-0 PMC6841727

[27] Privé F, Vilhjálmsson BJ, Aschard H, Blum MG. Making the most of clumping and thresholding for polygenic scores. Am J Hum Genet. 2019;105(6):1213–21. 31761295 10.1016/j.ajhg.2019.11.001 PMC6904799

[28] Privé F, Arbel J, Vilhjálmsson BJ. LDpred2: better, faster, stronger. Bioinformatics. 2020;36(22–23):5424–31. 10.1093/bioinformatics/btaa1029 PMC8016455 33326037

[29] Escott-Price V, Myers A, Huentelman M, Shoai M, Hardy J. Polygenic risk score analysis of Alzheimer’s disease in cases without APOE4 or APOE2 alleles. J Prev Alzheimer’s disease. 2019;6(1):16–9. 30569081 10.14283/jpad.2018.46 PMC6399990

[30] Gunter NB, Gebre RK, Graff-Radford J, Heckman MG, Jack CR Jr, Lowe VJ, et al. Machine learning models of polygenic risk for enhanced prediction of Alzheimer disease endophenotypes. Neurology: Genet. 2024;10(1):e200120. 10.1212/NXG.0000000000200120 PMC10798228 38250184

[31] Hermes S, Cady J, Armentrout S, O’Connor J, Holdaway SC, Cruchaga C, et al. Epistatic features and machine learning improve Alzheimer’s disease risk prediction over polygenic risk scores. J Alzheimer’s Disease. 2024;99(4):1425–40. 38788065 10.3233/JAD-230236 PMC11284654

[32] Li F, Xie S, Cui J, Li Y, Li T, Wang Y, et al. Polygenic risk score reveals genetic heterogeneity of Alzheimer’s disease between the Chinese and European populations. J Prev Alzheimers Dis. 2024;11(3):701–9. 10.14283/jpad.2024.29 38706286

[33] Escott-Price V, Myers AJ, Huentelman M, Hardy J. Polygenic risk score analysis of pathologically confirmed Alzheimer disease. Ann Neurol. 2017;82(2):311–4. 28727176 10.1002/ana.24999 PMC5599118

[34] Hebert LE, Scherr PA, Beckett LA, Albert MS, Pilgrim DM, Chown MJ, et al. Age-specific incidence of Alzheimer’s disease in a community population. JAMA. 1995;273(17):1354–9. 7715060

[35] Escott-Price V, Schmidt KM. Pitfalls of predicting age‐related traits by polygenic risk scores. Ann Hum Genet. 2023;87(5):203–9. 37416935 10.1111/ahg.12520 PMC10952323

[36] Sando SB, Melquist S, Cannon A, Hutton ML, Sletvold O, Saltvedt I, et al. APOE ε4 lowers age at onset and is a high risk factor for Alzheimer’s disease; A case control study from central Norway. BMC Neurol. 2008;8:1–7. 18416843 10.1186/1471-2377-8-9 PMC2375917

[37] Palmqvist S, Tideman P, Mattsson-Carlgren N, Schindler SE, Smith R, Ossenkoppele R, et al. Blood biomarkers to detect Alzheimer disease in primary care and secondary care. JAMA. 2024;332(15):1245–57. 39068545 10.1001/jama.2024.13855 PMC11284636

[38] Lee LY, Vaghari D, Burkhart MC, Tino P, Montagnese M, Li Z, et al. Robust and interpretable AI-guided marker for early dementia prediction in real-world clinical settings. EClinicalMedicine. 2024;74:102725. 10.1016/j.eclinm.2024.102725. 10.1016/j.eclinm.2024.102725 PMC11701481 39764178

[39] Khera AV, Chaffin M, Aragam KG, Haas ME, Roselli C, Choi SH, et al. Genome-wide polygenic scores for common diseases identify individuals with risk equivalent to monogenic mutations. Nat Genet. 2018;50(9):1219–24. 30104762 10.1038/s41588-018-0183-z PMC6128408

[40] Eraslan G, Avsec Ž, Gagneur J, Theis FJ. Deep learning: new computational modelling techniques for genomics. Nat Rev Genet. 2019;20(7):389–403. 30971806 10.1038/s41576-019-0122-6

[41] Eraslan G, Simon LM, Mircea M, Mueller NS, Theis FJ. Single-cell RNA-seq denoising using a deep count autoencoder. Nat Commun. 2019;10(1):390. 30674886 10.1038/s41467-018-07931-2 PMC6344535

[42] Vivian-Griffiths T, Baker E, Schmidt KM, Bracher‐Smith M, Walters J, Artemiou A, et al. Predictive modeling of schizophrenia from genomic data: Comparison of polygenic risk score with kernel support vector machines approach. Am J Med Genet Part B: Neuropsychiatric Genet. 2019;180(1):80–5. 10.1002/ajmg.b.32705 PMC6492016 30516002

[43] Romagnoni A, Jégou S, Van Steen K, Wainrib G, Hugot J-P. Comparative performances of machine learning methods for classifying Crohn Disease patients using genome-wide genotyping data. Sci Rep. 2019;9(1):10351. 31316157 10.1038/s41598-019-46649-z PMC6637191

[44] Wilkinson T, Ly A, Schnier C, Rannikmäe K, Bush K, Brayne C, et al. Identifying dementia cases with routinely collected health data: a systematic review. Alzheimer’s Dement. 2018;14(8):1038–51. 29621480 10.1016/j.jalz.2018.02.016 PMC6105076

[45] Escott-Price V, Myers A, Huentelman M, Shoai M, Hardy J. Polygenic risk score analysis of Alzheimer’s disease in cases without APOE4 or APOE2 alleles. J Prev Alzheimer’s disease. 2019;6:16–9. 30569081 10.14283/jpad.2018.46 PMC6399990

[46] Chaudhury S, Brookes KJ, Patel T, Fallows A, Guetta-Baranes T, Turton JC, et al. Alzheimer’s disease polygenic risk score as a predictor of conversion from mild-cognitive impairment. Translational psychiatry. 2019;9(1):154. 31127079 10.1038/s41398-019-0485-7 PMC6534556

[47] Kikuchi M, Miyashita A, Hara N, Kasuga K, Saito Y, Murayama S, et al. Polygenic effects on the risk of Alzheimer’s disease in the Japanese population. Alzheimers Res Ther. 2024;16(1):45. 38414085 10.1186/s13195-024-01414-x PMC10898021

[48] Trares K, Stocker H, Stevenson-Hoare J, Perna L, Holleczek B, Beyreuther K, et al. Comparison of subjective cognitive decline and polygenic risk score in the prediction of all-cause dementia, Alzheimer’s disease and vascular dementia. Alzheimers Res Ther. 2024;16(1):188. 39160600 10.1186/s13195-024-01559-9 PMC11331600

[49] Hou T, Liu K, Fa W, Liu C, Zhu M, Liang X, et al. Association of polygenic risk scores with Alzheimer’s disease and plasma biomarkers among Chinese older adults: A community-based study. Alzheimer’s & Dementia. 2024. 10.1002/alz.13924 PMC11485307 39171679

[50] Yu C, Ryan J, Orchard SG, Robb C, Woods RL, Wolfe R, et al. Validation of newly derived polygenic risk scores for dementia in a prospective study of older individuals. Alzheimer’s Dement. 2023;19(12):5333–42. 37177856 10.1002/alz.13113 PMC10640662

[51] Stevenson-Hoare J, Heslegrave A, Leonenko G, Fathalla D, Bellou E, Luckcuck L, et al. Plasma biomarkers and genetics in the diagnosis and prediction of Alzheimer’s disease. Brain. 2023;146(2):690–9. 35383826 10.1093/brain/awac128 PMC9924904

[52] Sariya S, Felsky D, Reyes-Dumeyer D, Lali R, Lantigua RA, Vardarajan B, et al. Polygenic risk score for Alzheimer’s disease in Caribbean Hispanics. Ann Neurol. 2021;90(3):366–76. 34038570 10.1002/ana.26131 PMC8435026

[53] Johansen M, Joensen S, Restorff M, Stórá T, Christy D, Gustavsson EK, et al. Polygenic risk of Alzheimer’s disease in the Faroe Islands. Eur J Neurol. 2022;29(8):2192–200. 35384166 10.1111/ene.15351

[54] Ikonnikova A, Morozova A, Antonova O, Ochneva A, Fedoseeva E, Abramova O, et al. Evaluation of the Polygenic Risk Score for Alzheimer’s Disease in Russian Patients with Dementia Using a Low-Density Hydrogel Oligonucleotide Microarray. Int J Mol Sci. 2023;24(19):14765. 37834213 10.3390/ijms241914765 PMC10572681

[55] Suh EH, Lee G, Jung S-H, Wen Z, Bao J, Nho K, et al. An interpretable Alzheimer’s disease oligogenic risk score informed by neuroimaging biomarkers improves risk prediction and stratification. Front Aging Neurosci. 2023;15:1281748. 37953885 10.3389/fnagi.2023.1281748 PMC10637854

[56] Jiao B, Xiao X, Yuan Z, Guo L, Liao X, Zhou Y, et al. Associations of risk genes with onset age and plasma biomarkers of Alzheimer’s disease: a large case–control study in mainland China. Neuropsychopharmacology. 2022;47(5):1121–7. 35001095 10.1038/s41386-021-01258-1 PMC8938514

