# Supplementary material for: Improved polygenic risk prediction for alzheimer’s disease and related dementias using deep learning: age and APOE-stratified analysis
Source: Alzheimers Res Ther. 2026 Mar 12;18:76. doi: 10.1186/s13195-026-02011-w (PMC13063846; doi:10.1186/s13195-026-02011-w)
Supplement: Supplementary file 1 — Supplementary Material 1. Supplementary Figure 1. Flow chart for selecting UK Biobank (UKB) participants. Participants were selected based on self-identified ancestry using Data-Field 22006. A total of 339,332 individuals without kinship were identified using Data-Field 22021. [file 13195_2026_2011_MOESM1_ESM.pdf]

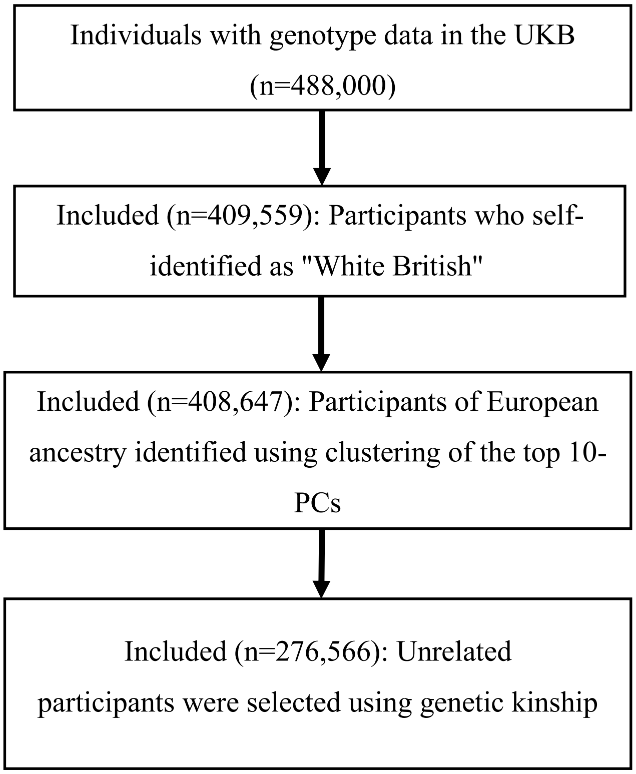

Supplementary Figure 1. A flow chart for selecting the UKB participants. The self-identified participants are based on the Data-Filed (22006) and 339,332 individuals without kinship were identified according to the Data-Field (22021).
