# Supplementary material for: Improved polygenic risk prediction for alzheimer’s disease and related dementias using deep learning: age and APOE-stratified analysis
Source: Alzheimers Res Ther. 2026 Mar 12;18:76. doi: 10.1186/s13195-026-02011-w (PMC13063846; doi:10.1186/s13195-026-02011-w)
Supplement: Supplementary file 2 — Supplementary Material 2. Supplementary Figure 2. Area under the ROC curve (AUC) for polygenic risk scores (PRSs) across age groups in the UK Biobank. AUC values for all PRSs were evaluated on the testing set (N = 92,188), stratified by age at baseline in 5-year intervals. [file 13195_2026_2011_MOESM2_ESM.pdf]

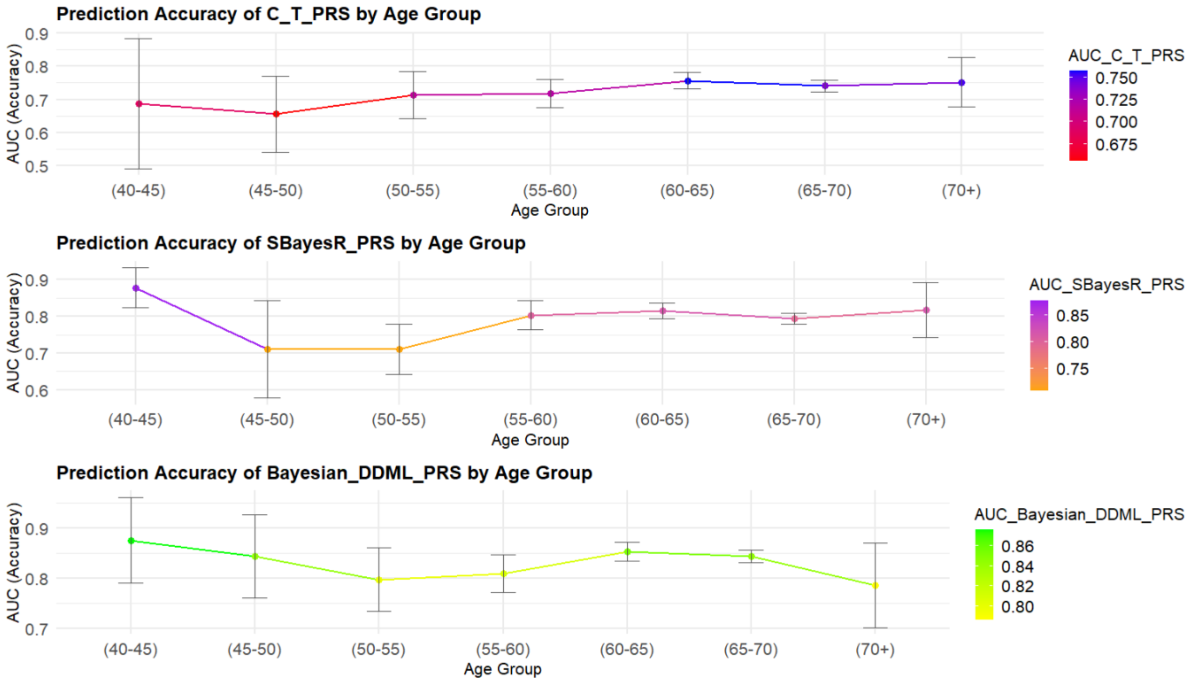

Supplementary Figure 2. Area under the ROC curve (AUC) for polygenic risk scores (PRSs) across age groups in the UK Biobank. AUC values for all PRSs were evaluated on the testing set (N = 92,188), stratified by age at baseline in 5-year intervals.
