# Supplementary material for: Improved polygenic risk prediction for alzheimer’s disease and related dementias using deep learning: age and APOE-stratified analysis
Source: Alzheimers Res Ther. 2026 Mar 12;18:76. doi: 10.1186/s13195-026-02011-w (PMC13063846; doi:10.1186/s13195-026-02011-w)
Supplement: Supplementary file 3 — Supplementary Material 3. Supplementary Figure 3. AUC for PRSs stratified by APOE-ε4 carrier status and age in the UK Biobank. Performance of all PRSs on the testing set (N = 92,188), stratified by APOE-ε4 carrier status and age at baseline. [file 13195_2026_2011_MOESM3_ESM.pdf]

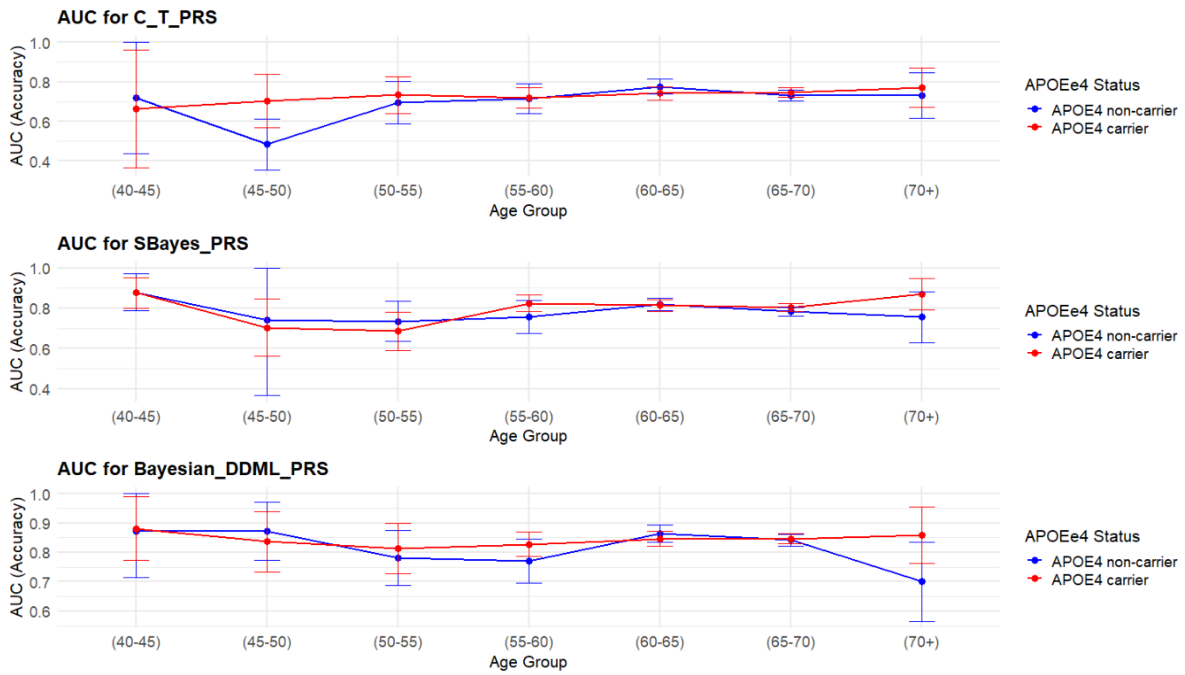

Supplementary Figure 3. AUC for PRSs stratified by *APOE-ε4* carrier status and age. Performance of all PRSs on the testing set (N = 92,188) of the UK Biobank, stratified by *APOE-ε4* carrier status and age at baseline.
