# Supplementary material for: Improved polygenic risk prediction for alzheimer’s disease and related dementias using deep learning: age and APOE-stratified analysis
Source: Alzheimers Res Ther. 2026 Mar 12;18:76. doi: 10.1186/s13195-026-02011-w (PMC13063846; doi:10.1186/s13195-026-02011-w)
Supplement: Supplementary file 4 — Supplementary Material 4. Supplementary Figure 4. AUC for PRS models by APOE-ε4 status and ADRD subtypes in the UK Biobank. AUC values for all PRS models on the testing set (N = 92,188), stratified by APOE-ε4 carrier status and Alzheimer’s Disease and Related Dementias (ADRD) subtypes. Subtypes include Alzheimer’s Disease (AD), vascular dementia, and other, unspecified, or mixed dementias. [file 13195_2026_2011_MOESM4_ESM.pdf]

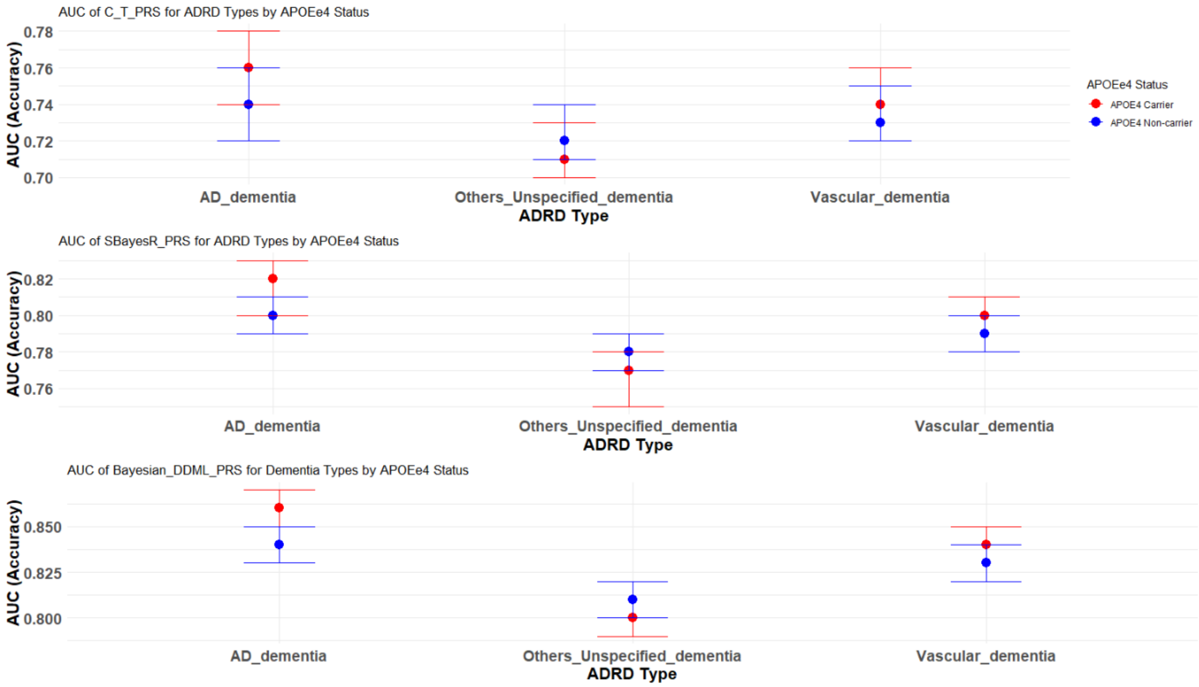


**Supplementary Figure 4.** AUC for PRS models by *APOE-ε4* status and ADRD subtypes in the UK Biobank. AUC values for all PRS models on the testing set (N = 92,188), stratified by *APOE-ε4* carrier status and Alzheimer's Disease and Related Dementias (ADRD) subtypes. Subtypes include Alzheimer's Disease (AD), vascular dementia, and other/unspecified or mixed dementias. The other/unspecified or mixed dementias category includes cases coded with ICD-10 diagnoses such as F03 (unspecified dementia), F02.8 (dementia in other diseases classified elsewhere), F09 (unspecified mental disorder due to brain damage), and G31.8 (other specified degenerative diseases of the nervous system).
