# Supplementary material for: Improved polygenic risk prediction for alzheimer’s disease and related dementias using deep learning: age and APOE-stratified analysis
Source: Alzheimers Res Ther. 2026 Mar 12;18:76. doi: 10.1186/s13195-026-02011-w (PMC13063846; doi:10.1186/s13195-026-02011-w)
Supplement: Supplementary file 5 — Supplementary Material 5. Supplementary Table 1. PRS-only (no covariates) discrimination for ADRD in the UK Biobank with and without the APOE region. Supplementary Table 2. Confusion matrix metrics (N) for the top performing PRS (i.e., DDML_PRS) across age groups at baseline on the whole sample (N= 276,566). Supplementary Table 3. The number of correctly classified individuals by the top performing PRS (i.e., DDML_PRS) in the UK Biobank (N=276,566) with prevalence of 0.48%. Supplementary Table 4. Comparison between the number (%) of correctly classified individuals (CC) by the top performing model (i.e., DDML_PRS) among APOE-ε4 carriers and non-carriers in the UK Biobank (N=276,566) and prevalence of 0.48% . Supplementary Table 5. The number of correctly classified individuals (CC) by the top performing PRS (i.e., DDML_PRS) between women and men in the UK Biobank (N=276,566) and prevalence of 0.48%. Supplementary Table 6. Comparison of predictive accuracy of PRS (AUC, 95% CI) for any dementia type or mild-cognitive impairment (MCI) prediction from this study and published studies [32, 33, 45–56]. Supplementary Table 7. Population summary of age at baseline, ADRD cases, follow-up period in ADRD cases, and age at diagnosis in the UK Biobank (N = 276,566, prevalence of ADRD = 0.48%). Supplementary Table 8. Model specification details for the DDML_PRS framework used in ADRD risk prediction. [file 13195_2026_2011_MOESM5_ESM.docx]

**Supplementary Table 1.** PRS-only (no covariates) discrimination for ADRD in the UK Biobank with and without the *APOE* region

| **PRS model** | **With *APOE* region (AUC, 95% CI)** | **Without *APOE* region (AUC, 95% CI)** | ***P*-value** |
| --- | --- | --- | --- |
| DDML_PRS, AUC (95% CI) | 0.6907 (0.68-0.70) | 0.6542 (0.65-0.67) | <0.001 |
| SBayesR_PRS, AUC (95% CI) | 0.6604 (0.65-0.67) | 0.6101 (0.60-0.62) | <0.001 |
| C+T_PRS, AUC (95% CI) | 0.6120 (0.60-0.62) | 0.5805 (0.57-0.59) | <0.001 |
| Logistic regression (LR), AUC (95% CI) | 0.5938 (0.58-0.60) | 0.5519 (0.54-0.56) | <0.001 |
| *P*-value (LR as a reference) | *P*< 1×10^-4^ | *P*<1×10^-5^ | - |

AUCs were computed on the independent test set using PRS as the only predictor (no age, sex, genetic principal components, or *APOE* genotype covariates). “With *APOE* region” indicates that variants within the predefined *APOE* window were retained during PRS construction; “Without *APOE* region” indicates that all variants within the *APOE* window (GRCh37/hg19: chr19: 44–46 Mb) were removed prior to PRS construction and the PRS was recomputed. P-values in the last column compare AUCs between “with” and “without” *APOE* region for the same PRS method (two-sided test of correlated ROC curves on the same test set, as described in Methods).

**Supplementary Table 2.** Confusion matrix metrics (N) for the top performing PRS (i.e., DDML_PRS) across age groups at baseline on the whole sample (N= 276,566)

| **Age at baseline** | **TP** | **FN** | **FP** | **TN** | **CC** | **N** | **% of CC** |
| --- | --- | --- | --- | --- | --- | --- | --- |
| (40-44) | 9 | 9,157 | 0 | 16,823 | 16,832 | 25,989 | 64.74% |
| (45-49) | 17 | 12,640 | 2 | 22,241 | 22,258 | 34,800 | 63.97% |
| (50-54) | 34 | 14,884 | 7 | 26,683 | 26,717 | 41,508 | 64.36% |
| (55-59) | 101 | 17,684 | 24 | 32,227 | 32,328 | 50,036 | 64.62% |
| (60-64) | 308 | 24,685 | 45 | 45,062 | 45,370 | 70,100 | 64.73% |
| (65-69) | 633 | 18,079 | 116 | 33,792 | 34,425 | 52,620 | 65.45% |
| (70-74) | 24 | 430 | 8 | 850 | 874 | 1,312 | 66.61% |

TP: True positive, FN: False negative, FP: False positive, TN: True negative, CC: Correctly classified individuals (TP+TN), N: Total Number of Individuals: A linear trend of percentage of CC was observed with age, the optimal threshold was determined using Youden index (sensitivity+specificity-1). The Cochran-Armitage trend test was used to test relationship between classification accuracy and age.

**Supplementary Table 3.** The number of correctly classified individuals by the top performing PRS (i.e., DDML_PRS) in the UK Biobank (N=276,566) with prevalence of 0.48%.

| **Age at baseline** | **Full model (TP+TN)** | **Null model (TP+TN)** | **DCC** |
| --- | --- | --- | --- |
| (40-44) | 16,832 | 16,823 | +9 |
| (45-49) | 22,258 | 22,241 | +17 |
| (50-54) | 26,717 | 26,683 | +34 |
| (55-59) | 32,328 | 32,227 | +101 |
| (60-64) | 45,370 | 45,062 | +308 |
| (65-69) | 34,425 | 33,792 | +633 |
| (70-74) | 874 | 850 | +24 |

DCC: Difference Correct Classification (represents the additional number of correctly classified individuals achieved by the PRS-informed model relative to the age-only null model), full model (PRS + age) and null model (age) in number. Full model AD risk= logit(B1*PRS+B2*age), null model = AD risk = logit(B1*age).

**Supplementary Table 4.** Comparison between the number (%) of correctly classified individuals (CC) by the top performing model (i.e., DDML_PRS) among *APOE-ε4* carriers and non-carriers in the UK Biobank (N=276,566) and prevalence of 0.48%

| **Age at baseline** | **CC in *APOE-ε4* carriers (%)** | **CC in *APOE-ε4* non-carriers (%)** | **DCC (%)** | ***P*-value** |
| --- | --- | --- | --- | --- |
| (40-44) | 62.50% | 62.41% | 0.09 % | 0.901 |
| (45-49) | 62.39% | 61.58% | 0.81 % | 0.211 |
| (50-54) | 62.62% | 62.23% | 0.39 % | 0.547 |
| (55-59) | 62.56% | 62.50% | 0.06 % | 0.595 |
| (60-64) | 62.77% | 62.72% | 0.05 % | 0.494 |
| (65-69) | 63.09% | 62.82% | 0.27 % | **0.048** |
| (70-74) | 66.47% | 64.71% | 1.76 % | 0.783 |

DCC: Difference of % of Correct Classification (CC) between *APOE-ε4* carriers and non-carriers, p-values were derived using a Z-test for CC proportions to compare the two independent groups (*APOE-ε4* -positive vs *APOE-ε4* -negative) in each age group.

**Supplementary Table 5.** The number of correctly classified individuals (CC) by the top performing PRS (i.e., DDML_PRS) between women and men in the UK Biobank (N=276,566) and prevalence of 0.48%.

| **Age at baseline** | **N of CC in women (%)** | **N of CC in men (%)** | **DCC (%)** | ***P*-value** |
| --- | --- | --- | --- | --- |
| (40-44) | 8858 (64.92%) | 7974 (64.59%) | 0.33 % | 0.579 |
| (45-49) | 12270 (64.04%) | 9988 (63.45%) | 0.59 % | 0.253 |
| (50-54) | 15045 (64.47%) | 11672 (63.88%) | 0.59 % | 0.216 |
| (55-59) | 17712 (64.38%) | 14616 (64.89%) | -0.51 % | 0.229 |
| (60-64) | 24086 (64.49%) | 21284 (64.99%) | -0.50 % | 0.171 |
| (65-69) | 16890 (65.43%) | 17535 (65.41%) | 0.02 % | 0.971 |
| (70-74) | 416 (67.10%) | 458 (66.18%) | 0.92 % | 0.727 |

DCC: Difference of % of Correct Classification (CC) proportion between women and men (N of women- N of men), P-values were derived using a Z-test for the proportions to compare the two independent groups (women vs men) in each age group.

**Supplementary Table 6.** Comparison of predictive accuracy of PRS (AUC, 95% CI) for any dementia type or mild-cognitive impairment (MCI) prediction from this study and published studies

| **Study** | **Data Source** | **Method/Meta-data/Risk factors** | **AUC (95% CI)** | **Median length of follow-up** | **Prevalence of AD** | **P-value** |
| --- | --- | --- | --- | --- | --- | --- |
| Our proposed method | UKB | Bayesian Variational Autoencoders (DDML_PRS) (PRS+Age+Sex+*APOE*) | 0.83 (0.83-0.85) | 9.19 years | 0.48% | Ref. |
| Escott-Price et al. (2019)(43) | IGAP | PRS with SNPs p≤0.001 | 0.74 (0.72-0.78) | - | 70% | <0.001 |
| Chaudhury et al. (2019)(44) | ICOS | PRSice-2+ Age+Sex | 0.82 | 3 years | 40% | NA |
| Kikuchi et al. (2024) (45) | NP and NA-ADNI | Clumping and Thresholding PRS | 0.71 (0.64-0.76) | - | 49% | <0.001 |
| Li F et al. (2024) (46) | A Chinese population | PRSnoAPOE + *APOE*+ Sex + Age | 0.83 (0.74-0.92) | - | 11.3% | NS |
| Trares K et al. (2024) (47) | ESTHER | PRS+ age+ education+ sex+ systolic blood pressure+BMI+ total cholesterol + physical activity | 0.80 (0.78-0.82) | 17 years | 7.6% | <0.01 |
| Hou T et al. (2024) (48) | MIND‐China and SYS‐AD | PRS+Age+Sex+Education | 0.80 (0.77-0.84) | 3.78 years | 3.7% | <0.01 |
| Yu C et al. (2023) (49) | ASPREE | non-genetic risk factors+ *APOE* + PRS-SBayesR + PRS-CS | 0.76 (0.74-0.78) | 6.5 years | 4.2% | <0.001 |
| Stevenson-Hoare et al. (2023) (50) | ADCC | Blood biomarkers+ sex+ *APOE* + PRS | 0.81 | - | 74% | NA |
| Sariya S et al. (2021) (51) | CH | CH-PRS + sex + age + *APOE-ϵ4* | 0.74 | - | 50% | NA |
| Johansen M et al. (2022) (52) | Faroese Alzheimer's project | Weighted_PRS/Kunkle | 0.80 (0.75-0.85) | 4 years | 52.2% | <0.01 |
| Ikonnikova et al. (2023) (53) | gerontological department of Mental-health Clinic No. 1 | Weighted_PRS/ Lambert JC /both social and genetic factors | 0.734 | - | 40% | NA |
| Suh EH et al. (2023) (54) | ADNI | Clumping and Thresholding PRS | 0.75 (0.70-0.80) | 5 years | 60.1% | <0.001 |
| Jiao B et al. (2022) (55) | Xiangya Hospital | PRSice-2 | 0.71 (0.69-0.72) | - | 33.1% | <0.001 |
| Escott-Price et al. (2017) (56) | IGAP | PRS with SNPs *P*≤0.5 | 0.86 | - | 63.4% | NA |

Our study (the first study) was considered as a reference for statistical comparison of AUCs with their 95% CIs using Wald-Z test, NA: Not reported, NS: Not statistically significant at level of 0.05 because no CI was reported in that study.

**Supplementary Table 7.** Population summary of age at baseline, ADRD cases, follow-up period in ADRD cases, and age at diagnosis in the UK Biobank (N = 276,566, prevalence of ADRD = 0.48%)

| **Age at baseline** | **N of individuals (%)** | **N of ADRD cases (%)** | **Follow up in ADRD cases (years)** | **Age at diagnosis (years)** |
| --- | --- | --- | --- | --- |
| (40-44) | 25,989 (9.4%) | 9 (0.68%) | 7.28 (1.59) | 47.76 (2.78) |
| (45-49) | 34,800 (12.6%) | 19 (1.43%) | 7.28 (3.90) | 52.68 (3.83) |
| (50-54) | 41,508 (15.03%) | 41 (3.09%) | 6.21 (1.74) | 58.20 (3.54) |
| (55-59) | 50,036 (18.09%) | 125 (9.42%) | 6.20 (2.26) | 63.41 (3.27) |
| (60-64) | 70,100 (25.37%) | 353 (26.62%) | 6.22 (2.49) | 68.51 (3.25) |
| (65-69) | 52,620 (19.03%) | 749 (56.38%) | 5.92 (2.84) | 73.40 (3.62) |
| (70-74) | 1,312 (0.47%) | 32 (2.41%) | 5.19 (1.55) | 75.43 (2.77) |

Follow-up and age at diagnosis are presented for Alzheimer’s disease and related dementias (ADRD) cases as median (IQR: Interquartile range).

**Supplementary Table 8.** Model specification details for the DDML_PRS framework used in ADRD risk prediction

| **Field** | **Value / Details** |
| --- | --- |
| **Model run ID** | DDML_PRS_final |
| **Input data** | Individual-level genotype data for 80 preselected ADRD-associated SNPs |
| **Encoder architecture** | Three fully connected layers (512 → 256 → 128 units) with ReLU activations |
| **Latent space** | 50-dimensional latent representation; encoder outputs mean and log-variance vectors; reparameterization trick applied |
| **Decoder architecture** | Symmetric mirror of encoder (128 → 256 → 512) to reconstruct genotype input |
| **PRS derivation** | Posterior mean of the latent variables aggregated to a single continuous scalar score (DDML_PRS) |
| **GWAS-informed priors** | Prior means and variances derived from UK Biobank–excluded GWAS summary statistics from Jansen et al. (2019); effect sizes (BETA) and standard errors used to define informative Bayesian priors |
| **Role of priors** | Priors incorporated through the Kullback–Leibler (KL) divergence term to regularize the latent space and limit overfitting |
| **KL annealing schedule** | Linear annealing of KL weight from 0 to 1 over the first 20 training epochs |
| **Training data usage** | Models trained on genotype data only; no phenotypic covariates included as model inputs |
| **Covariate handling** | Age, sex, genetic principal components, and *APOE* genotype (where indicated) included only in downstream regression and survival models |
| **Hyperparameter specification** | Architecture and training hyperparameters pre-specified a priori based on prior VAE applications in large-scale genomics; no hyperparameter tuning performed on the test set |
| **Regularization strategy** | Bayesian GWAS-informed priors and early stopping based on validation ELBO (patience = 10 epochs) |
| **Data splitting and leakage control** | Prespecified 2:1 train/test split with identical ADRD case proportion; 10% of training data held out for internal validation; independent test set used only for final evaluation |

This table summarizes the architecture and configuration of the Bayesian variational autoencoder–based polygenic risk score (DDML_PRS) model used for ADRD risk prediction. It details the encoder–decoder structure, latent space design, derivation of the continuous PRS, incorporation of UK Biobank–excluded GWAS-informed Bayesian priors, Kullback–Leibler (KL) divergence annealing strategy, and safeguards implemented to prevent data leakage. Covariates were incorporated exclusively in downstream regression and survival models, not within the deep learning architecture.
